# Histology of 24 organs from Asian elephant calves (*Elephas maximus*)

**DOI:** 10.7717/peerj.4947

**Published:** 2018-06-14

**Authors:** Chatchote Thitaram, Pitchaya Matchimakul, Wanpitak Pongkan, Wasan Tangphokhanon, Raktham Maktrirat, Jaruwan Khonmee, Anucha Sathanawongs, Piyamat Kongtueng, Korakot Nganvongpanit

**Affiliations:** 1 Center of Excellence in Elephant and Wildlife Research, Faculty of Veterinary Medicine, Chiang Mai University, Chiang Mai, Thailand; 2 Center of Excellence in Veterinary Biosciences, Department of Veterinary Biosciences and Public Health, Faculty of Veterinary Medicine, Chiang Mai University, Chiang Mai, Thailand; 3 Central Laboratory, Faculty of Veterinary Medicine, Chiang Mai University, Chiang Mai, Thailand

**Keywords:** Elephant, Histology, Microanatomy, Tissue, Organ

## Abstract

**Background:**

Elephants are the largest and heaviest living terrestrial animals, but information on their histology is still lacking. This study provides a unique insight into the elephant’s organs and also provides a comparison between juvenile Asian elephants and adult Asian elephants or other species. Here we report on the histological structure of 24 organs, including the skin, brain (cerebrum, cerebellar hemisphere, vermis, thalamus, midbrain), spinal cord, sciatic nerve, striated skeletal muscle, cardiac muscle, bone (flat bone and long bone), cartilage (hyaline cartilage and fibrocartilage), heart (right atrium, right ventricle), blood vessels (aorta, pulmonary artery and caudal vena cava), trunk, trachea, lung, tongue, esophagus, stomach, small intestine (duodenum, jejunum, ileum), large intestine (cecum, colon, rectum), liver and pancreas, kidney, ovary, uterus (body and horn) and spleen of two juvenile Asian elephants.

**Methods:**

Tissue sections were stained with Harris’s hematoxylin and eosin Y.

**Results:**

While almost all structures were similar to those of other species or adult elephants, some structures were different from other mammalian species, such as: plexiform bone was found in flat bone only; a thin trachealismuscle was observed in the trachea; and no serous or mucinous glands were found in the submucosa of the trachea.

**Discussion:**

Histological information from various organs can serve as an important foundation of basal data for future microanatomical studies, and help in the diagnosis and pathogenesis in sick elephants or those with an unknown cause of death.

## Introduction

Elephants are the largest land animals and consist of two extant genera within the family Elephantidae: *Elephas* (Asian elephant; *Elephas maximus*) and *Loxodonta* (African elephant; *Loxodonta africana)*. The organ structure (macroanatomy and histology) as well as the physiology of this animal is often regarded as miraculous. Scientists want to know what those organs look like and how they function. However, studying the body structure and organ function in elephants is not easy. They cannot be kept and fed in a research facility like some other animals, and most are living in the wild, far away from the laboratory. Moreover, elephants are classified as threatened or endangered species. For these reasons, studies on the body structure and organ function of elephants has been limited, and even studies on one, two or three animals are still valuable (Ahasan et al., 2016; Egger et al., 2008; Meyer, Weissengruber & Busche, 2010; Papageorgopoulou, Link & Ruhli, 2015; Spearman, 1970; Van Aswegen et al., 1994, 1996).

Organ histology records patterns of growth and provides information on the biology, evolution and physiology of life, but little is known about the histology of elephant organs. Out of all the organs in the elephant body, a few organs have been studied for their histological structure; for examples: testis (Johnson & Buss, 1967; Jones & Holt, 1981), esophagus (Van Aswegen et al., 1994), stomach (Indu et al., 2014; Van Aswegen et al., 1994), intestine (Van Aswegen et al., 1996), adrenal gland (Kramer, Teixeira & Hattingh, 1991), skin (Spearman, 1970), temporal gland (Meyer, Weissengruber & Busche, 2010), articular cartilage (Egger et al., 2008) and bone (Curtin et al., 2012; Nganvongpanit et al., 2017b). Moreover, most studies have been done on African elephants (Curtin et al., 2012; Egger et al., 2008; Johnson & Buss, 1967; Jones & Holt, 1981; Kramer, Teixeira & Hattingh, 1991; Spearman, 1970; Van Aswegen et al., 1994, 1996), while studies on Asian elephants have been limited (Curtin et al., 2012; Indu et al., 2014; Nganvongpanit et al., 2017b).

Until now, scientists and anatomists still question whether the histology of the largest terrestrial tetrapod in the world is similar or different from that of other mammalian species. The aim of this study was to describe the microanatomy of 24 organs from the juvenile Asian elephant. This information could help us better understand the distinctive organ histology of elephants, in particular the juvenile stage.

## Materials and Methods

### Sample collection

Two juvenile Asian elephants were included in the study: a 2-year-old Asian elephant weighing 400 kg; and a 2-year and 9-month-old Asian elephant weighing 600 kg (born in October 2014, died from unknown causes) (Supplemental Material S1). Necropsy results on the first calf found some focal hemorrhage in some internal organs, while no pathological lesions were found on organs in the second calf. Hence, these juvenile elephants were deemed fit to serve as the subjects of our microanatomy study (among samples taken from the first calf, only parts without gross morphology were selected). Tissue samples were collected from the carcass within 12 h after death and placed in 10% neutral buffered formalin. A total of 40 tissues from nine different bodily systems were acquired, including:

1. *Integument system:* skin (from ear)
2. *Nervous system:* cerebrum, cerebellar hemisphere, vermis, thalamus, midbrain, spinal cord and sciatic nerve
3. *Muscular system:* striated skeletal muscle and cardiac muscle
4. *Skeletal system:* bone (long bone from the humerus and flat bone from the parietal bone) and cartilage (hyaline cartilage from the femoral head and fibrocartilage from the meniscus)
5. *Cardiovascular system:* heart (right atrium, right ventricle), aorta, pulmonary artery and caudal vena cava
6. *Respiratory system:* trunk (inner), trachea (lower part), lung (proximal, distal)
7. *Gastrointestinal system:* tongue, esophagus, stomach (cardia, fundus, pylorus), small intestine (duodenum, jejunum, ileum), large intestine (cecum, colon, rectum), liver and pancreas
8. *Urinary system:* kidney
9. *Reproductive system:* ovary, uterine horn and uterine body
10. *Lymphatic system:* spleen

According to the Animals for Scientific Purposes Act, B.E. 2558 (2015), since a part of this experiment was performed on an elephant carcass from a private owner during the diagnosis procedure for the cause of death, no ethical approval was required for this study and confirmed by the Animal Ethics Committee, Faculty of Veterinary Medicine, Chiang Mai University (Licence number U1006312558). However, the owner allowed the research team to take a sample of skin for this study.

### Histological study

Tissues were fixed in 10% neutral buffered formalin for 24 h; bone tissue was then decalcified with 10% nitric acid for 8 h. The specimens were cut into 1 mm pieces, placed in plastic cassettes and then processed in 10% formalin for 1 h (two changes), 95% ethanol for 1 h (three changes), absolute isopropyl alcohol for 1 h (two changes), xylene for 1 h (two changes), and Paraplast for 1 h (three changes). The tissues were then embedded in paraffin and cut into 5 µm sections.

Sections were deparaffinized in xylene and rehydrated through a series of alcohols to water. Tissue sections were stained with Harris’s hematoxylin for 5 min and washed under running tap water for 5 min; differentiated in 1% acid alcohol (1% hydrochloric acid in 70% ethanol) for 5 s and washed under tap water for 5 min; dipped in saturated lithium carbonate solution for 5 s and washed under tap water for 5 min; and stained with 1% eosin Y for 3 min and washed under running tap water for 5 min. The sections were then dehydrated through a graded ethanol series, cleared in xylene, and mounted in Permount. Individual sections were observed using a compound light microscope (Olympus BX53; Olympus Corporation, Tokyo, Japan).

## Results

### Integument system

**Figure 1:**
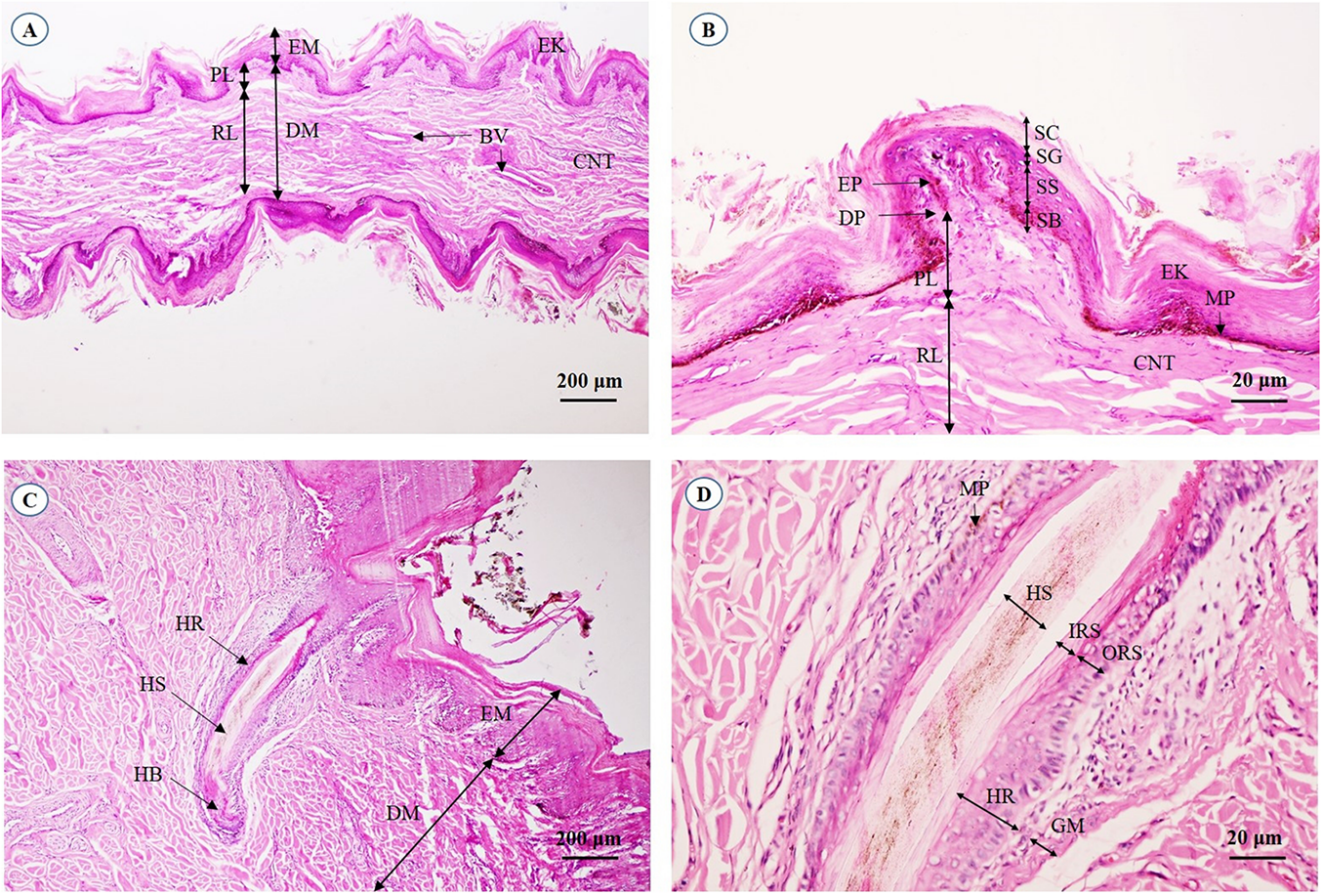
Light microscopymicrographs at different magnifications of the aural skin (A, B) and hair follicle (C, D). Study sites: BV, blood vessel; CNT, connective tissue; DM, dermis; DP, dermal papilla; EK, epidermal keratinocyte; EM, epidermis; EP, epidermal peg; GM, glassy membrane; HB, hair bulb; HR, hair root; HS, hair shaft; IRS, inner root sheath; MP, melanin pigment; ORS, outer root sheath; PL, papillary layer; RL, reticular layer; SB, stratum basale; SC, stratum corneum; SG, stratum granulosum; SS, stratum spinosum. Hematoxylin and eosin staining.

A cross-sectional area of the aural cutis (Fig. 1A) shows the skin divided into the epidermis and dermis. The epidermal layer without the stratum lucidum was observed, whereas numerous melanin-producing melanocytes were found in the stratum basale and stratum spinosum. The aural corium contained two sublayers, the stratum papillary and stratum reticular. The primary and secondary dermal papillae protruded interdigitally into the underside of the epidermis (Fig. 1B). High magnification of the cross-sectional area confirmed the absence of sudoriferous and sebaceous glands. Moreover, no arrector pili muscles were associated with the hair follicles (Figs. 1C and 1D).

### Nervous system

**Figure 2:**
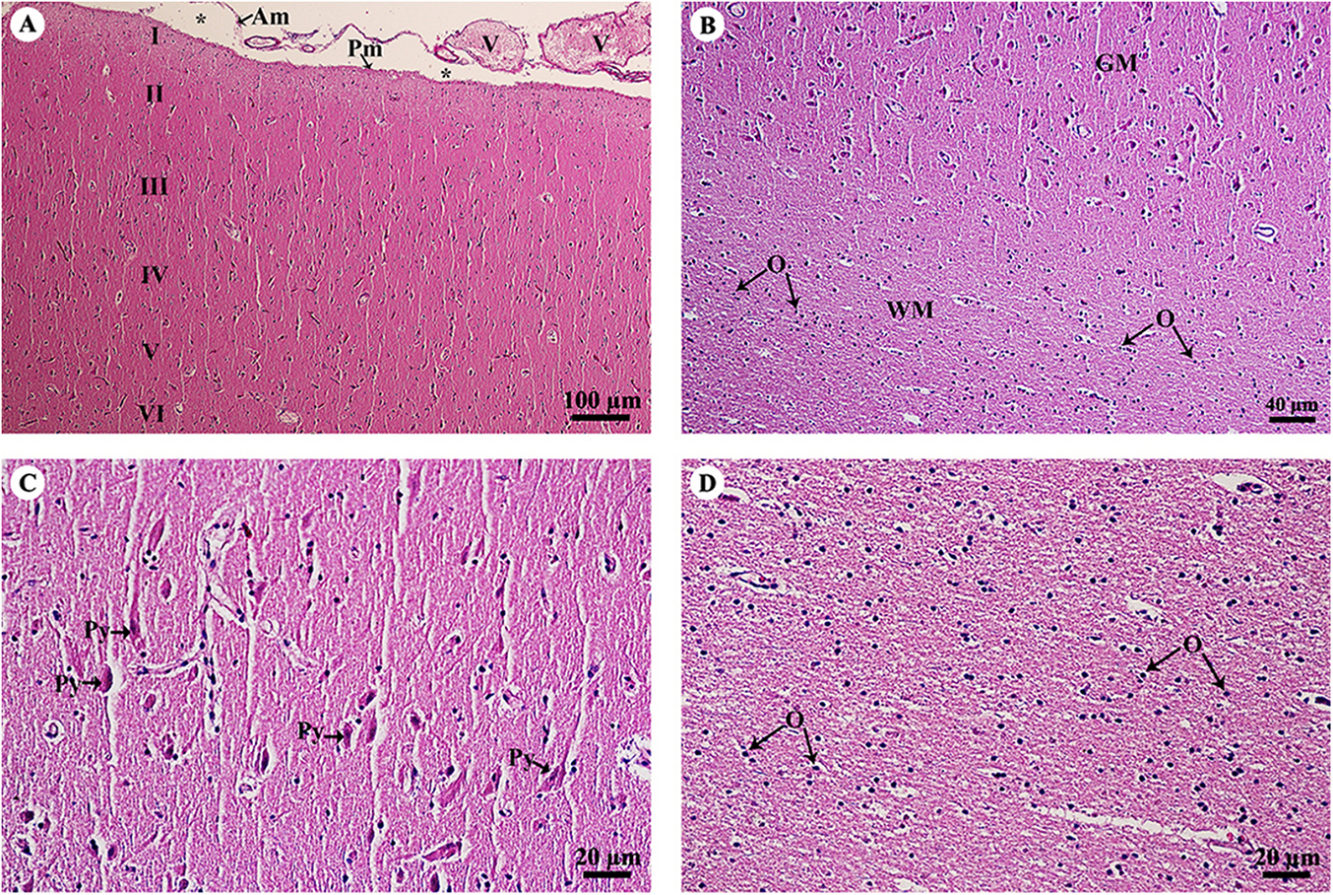
Light microscopy micrographs at different magnifications of the cerebrum (A–D). Study sites: Asterisksmark subarachnoid space; I, plexiform (molecular) layer; II, outer granular layer; III, pyramidal cell layer; IV, inner granular layer; V, ganglionic layer; VI, multiform cell layer; Am, arachnoid mater; GM, grey matter; O, oligodendrocyte; Pm, pia mater; Py, pyramidal cell; V, blood vessels; WM, white matter. Hematoxylin and eosin staining.

The cerebrum was covered with meninges, including the arachnoid mater and a thin layer of pia mater. Many small and large meningeal vessels were observed within the arachnoid mater. The subarachnoid space contained cerebrospinal fluid. The cerebral cortex (Fig. 2A) was composed of six layers (I–VI, from the cortex to the medulla) with different sizes, morphologies and types of neurons. Cortical neurons and glial cells were numerous in the cerebral gray matter but the density decreased at the junction of gray and white matter (Fig. 2B). In layer V of the cerebral cortex (Fig. 2C), the pyramidal cells were readily identified. Cerebral white matter (Fig. 2D) consisted of myelinated nerve fibers and glial cells. Oligodendrocytes were abundant and arranged in a column.

**Figure 3:**
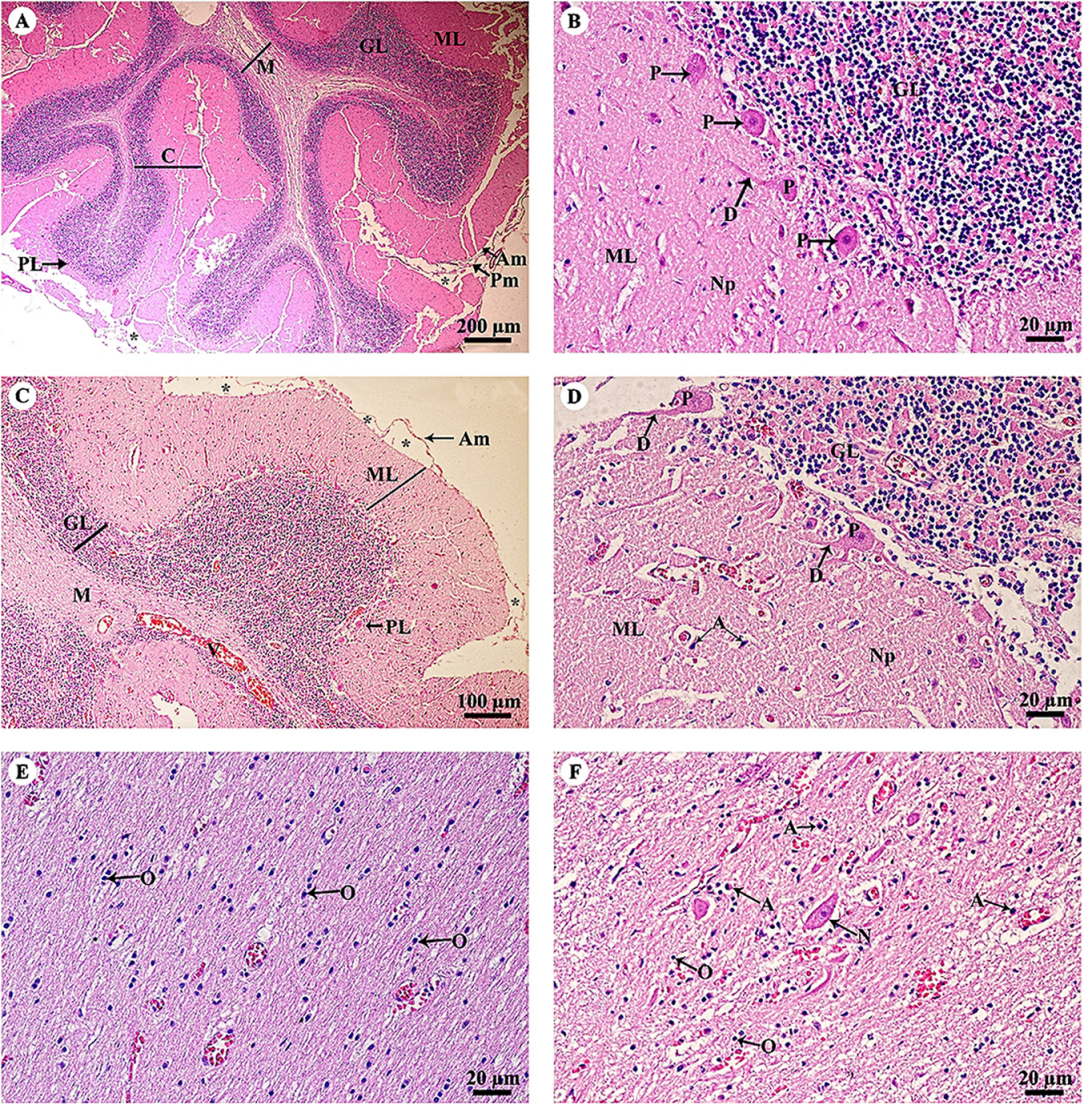
Light microscopy micrographs at different magnifications of the cerebellar hemisphere (A, B) and cerebellar vermis (C–F). Study sites: Asterisksmark subarachnoid space; A, astrocyte; Am, arachnoid mater; C, cerebellar cortex; D, dendrite; GL, granular layer; M, cerebellar medulla; ML, molecular layer; N, Neurone; Np, Neuropil; O, oligodendrocyte; P, Purkinje cells; PL, Purkinje layer; Pm, pia mater; V, blood vessels. Hematoxylin and eosin staining.

The cerebellar hemisphere (Fig. 3A) consisted of gray matter, or cerebellar cortex, and white matter, or cerebellar medulla. The cerebellar cortex was divided into three layers: the outer molecular layer, the Purkinje cell layer and the inner granular cell layer. In the Purkinje cell layer (Fig. 3B), large neurons named Purkinje cells were prominent, sending dendrites up into the outer molecular layer. The inner granular cell layer is highly cellular. The cerebellar vermis (Figs. 3C–3F) is the middle lobe of the cerebellum; the cortex layers (Fig. 3C) and cellular components (Fig. 3D) are similar to those of the cerebellar hemisphere. Cerebellar white matter (Fig. 3E) contained myelinated axons with numerous oligodendroglia. Deep cerebellar nuclei (Fig. 3F), probably the fastigial nucleus, were situated deep in the vermis. Glial cells, including astrocytes, were located adjacent to the vessels, and oligodendrocytes were commonly found.

**Figure 4:**
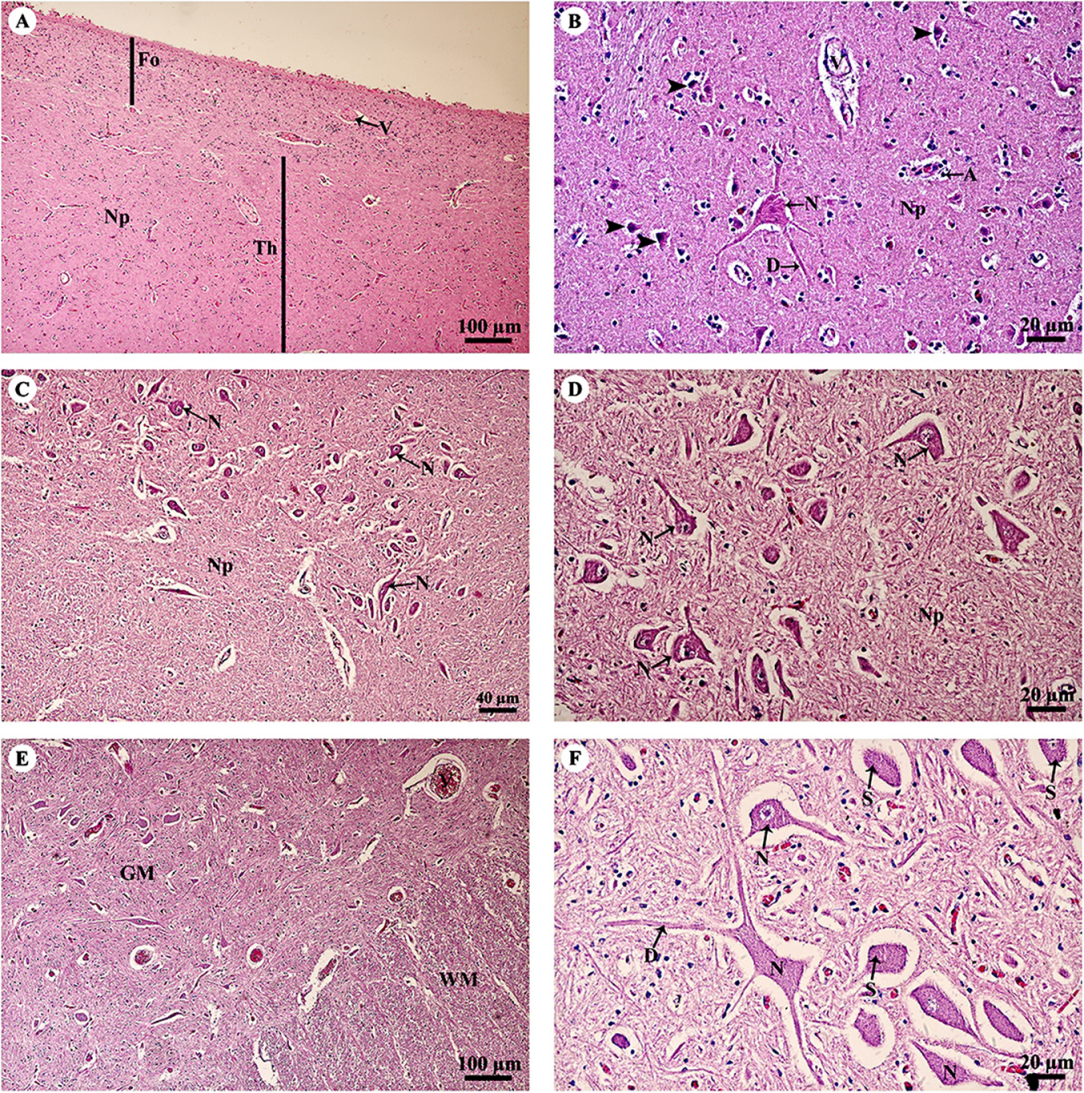
Light microscopy micrographs at different magnifications of the thalamus (A, B), midbrain (C, D) and spinal cord (E, F). Study sites: A, astrocyte; D, dendrite; Fo, fornix; GM, grey matter; N, Neurone; Np, Neuropil; S, Nissl substance; Th, thalamus; V, blood vessels; WM, white matter. Hematoxylin and eosin staining.

The thalamus (Fig. 4A) was covered with the fornix, comprised of layers of axons from the hippocampus. The thalamus contained typical basal nuclei with a loose distribution of neuron cell bodies. Large, distinct neurons formed the neuropil background (Fig. 4B). The midbrain (Figs. 4C and 4D) contained numerous multipolar neurons or substantia nigra. The spinal cord comprised central gray matter and peripheral white matter. A cross section of the ventral horn of the cervical spinal cord (Fig. 4E) displayed numerous neurons in the gray matter and extensive nerve fibers in the white matter. Neurons in the ventral horn (Fig. 4F) had multipolar morphology and are classified as alpha motor neurons.

**Figure 5:**
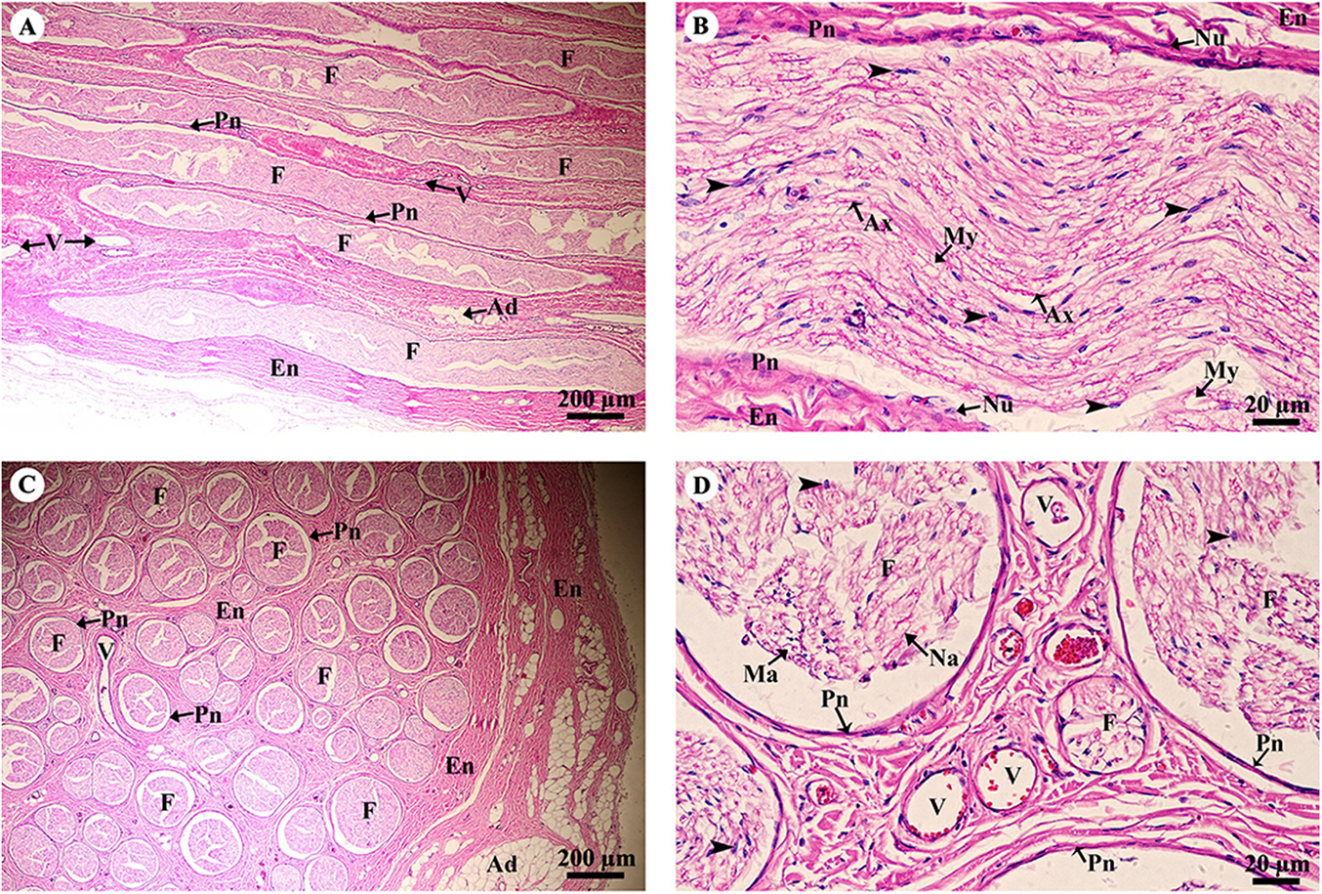
Light microscopy micrographs at different magnifications of the sciatic nerve, longitudinal section (A, B) and cross section (C, D). Study sites: Arrowheadspoint at Schwann cell nuclei; Ad, adipose tissue; Ax, axon; En, epineurium; F, fascicle; Ma, myelinated axon; My, myelin sheet; Na, non-myelinated axon; Nu, perineurial cell nuclei; Pn, perineurium; V, blood vessels.Hematoxylin and eosin staining.

A longitudinal section of the sciatic nerve (Fig. 5A) illustrates nerve fascicles surrounded by connective tissue sheaths. The perineurium is wrapped around the fascicle, while the epineurium surrounds the entire nerve and multiple fascicles. Blood vessels course within the epineurium and adipose tissue is found. At higher magnification (Fig. 5B), axons can clearly be seen surrounded by myelin sheaths produced by Schwann cells. Perineurial cell nuclei are found within the perineurium. A cross section of the sciatic nerve (Fig. 5C) shows numerous fascicles and very thick epineurium. The sciatic nerve is a mixed nerve; each fascicle contains myelinated and non-myelinated axons (Fig. 5D).

### Skeletal system

**Figure 6:**
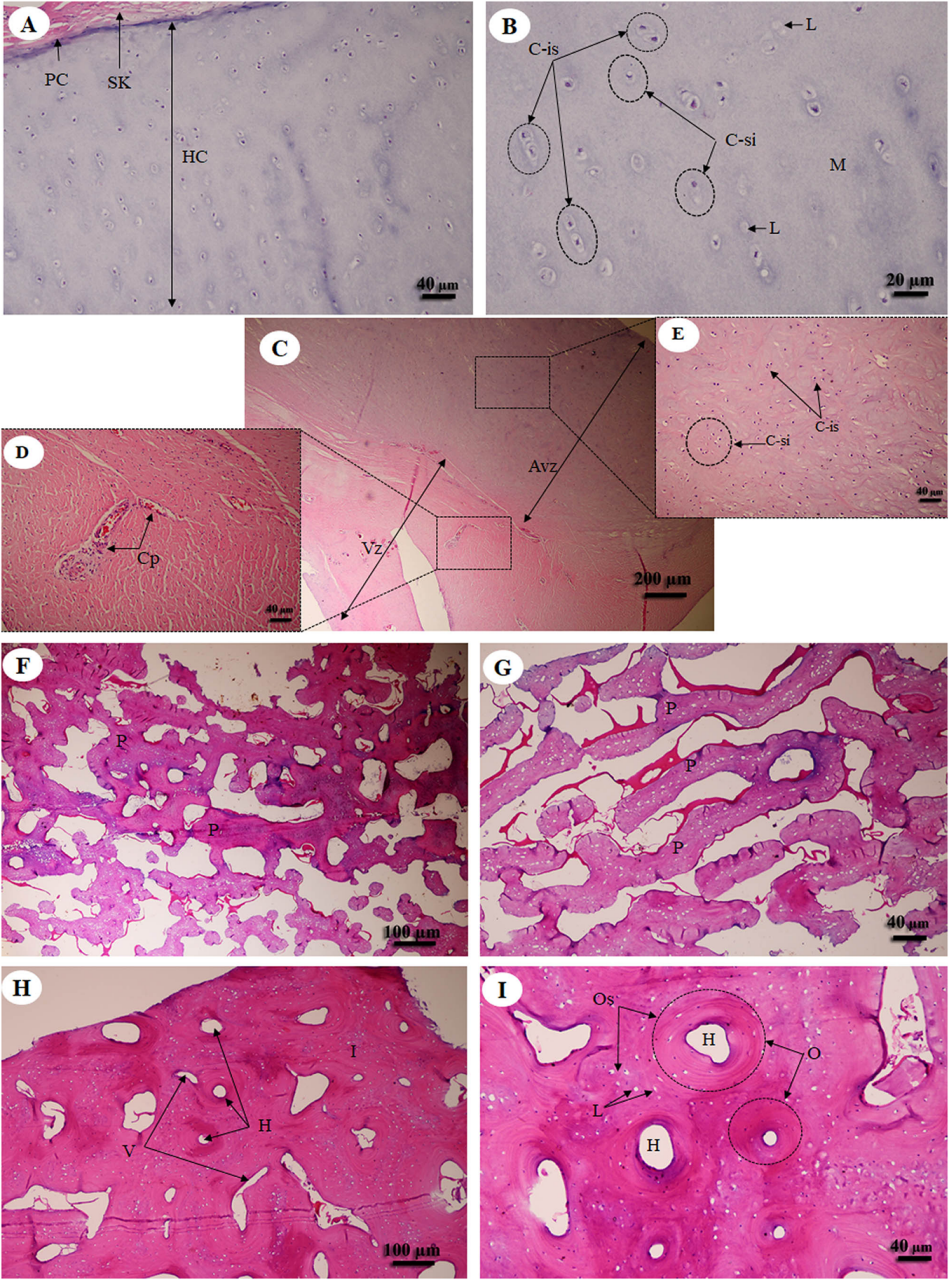
Low and high magnification of histological sections of hyaline cartilage from articular cartilage of femoral head (A, B), fibrocartilage from meniscus (C) with vascular (D) and avascular zone (E), flat bone from parietal bone (F, G) and long bone from cranial mid-shaft of right humerus (H, I). Study sites: Avz, avascular zone; C-is, isogenous group of chondrocytes; C-si, single chondrocyte; H, Haversian canal; HC, hyaline cartilage; I, interstitial lamella; L, lacuna; M, matrix; O, osteon; Os, osteocyte; P, plexiform bone; PC, perichondrium; SK, striated muscle; V, Volkmann’s canal; Vz, vascular zone. Hematoxylin and eosin staining.

Hyaline cartilage (Figs. 6A and 6B) was distributed throughout the homogeneous ground substance, or matrix. Ovoid spaces called lacunae contained mature cartilage cells, chondrocytes. Cartilage cells in the matrix were observed either singly or in isogenous groups, but were predominantly single chondrocytes. Fibrocartilage (Fig. 6C) also contained chondrocytes, either singly or in isogenous groups, but was dominated by single chondrocytes. Fibrocartilage from the meniscus was divided into two parts, vascular (Fig. 6D) and avascular zones (Fig. 6E). Compact bone from flat (Figs. 6F and 6G) and long bone (Figs. 6H and 6I) showed differences; compact bone from flat bone was woven (immature) bone with plexiform bone structure, but long bone was lamellar (mature) bone. Compact bone from long bone (Figs. 6F and 6G) presented osteons with varied size and shape, from circular to oval. Immature osteons were predominant, while mature osteons, plexiform bone and osteon banding were not found.

### Muscular system

**Figure 7:**
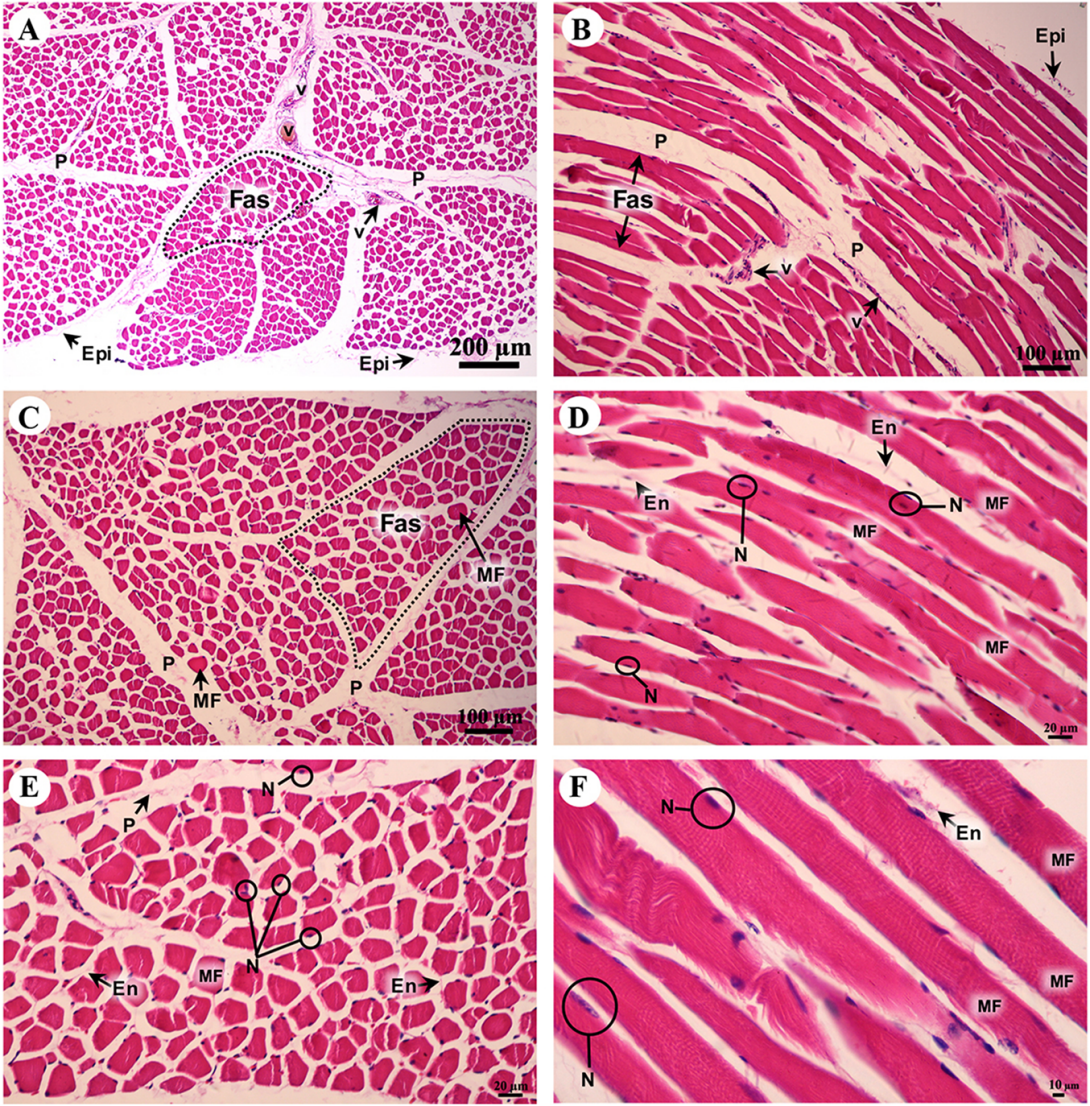
Low and high magnification of histological transverse sections (A, C, E) and longitudinal sections (B, D, F) of striated muscle from quadriceps femoris muscle. Study sites: Epi, epimysium; P, perimysium; En, endomysium; Fas, fascicle; MF, muscle fiber; N, nucleus; V, blood vessel. Hematoxylin and eosin staining.

A cross sectional area of striated muscle shows that muscle groups were covered by epimysium (Fig. 7A), whereas the muscle fascicle, a group of muscle fibers, was separated by perimysium (Figs. 7A and 7C). Blood vessels could be seen in this connective tissue (Fig. 7A). In addition, muscle fibers were separated by the endomysium which covered each individual muscle fiber itself, whereas the nucleus was located at the lateral border of the muscle fiber and beneath the sarcolemma (Figs. 7D and 7F). Moreover, a longitudinal section demonstrated the epimysium which covered the muscle and muscle fascicle (Fig. 7B). Blood vessels were found in the perimysium, as in the cross-sectional area, whereas the muscle fibers were also covered and separated by endomysium (Figs. 7B, 7D and 7F). Moreover, dark A bands and light I bands could be seen in some muscle fibers in this longitudinal section (Fig. 7F).

### Cardiovascular system

**Figure 8:**
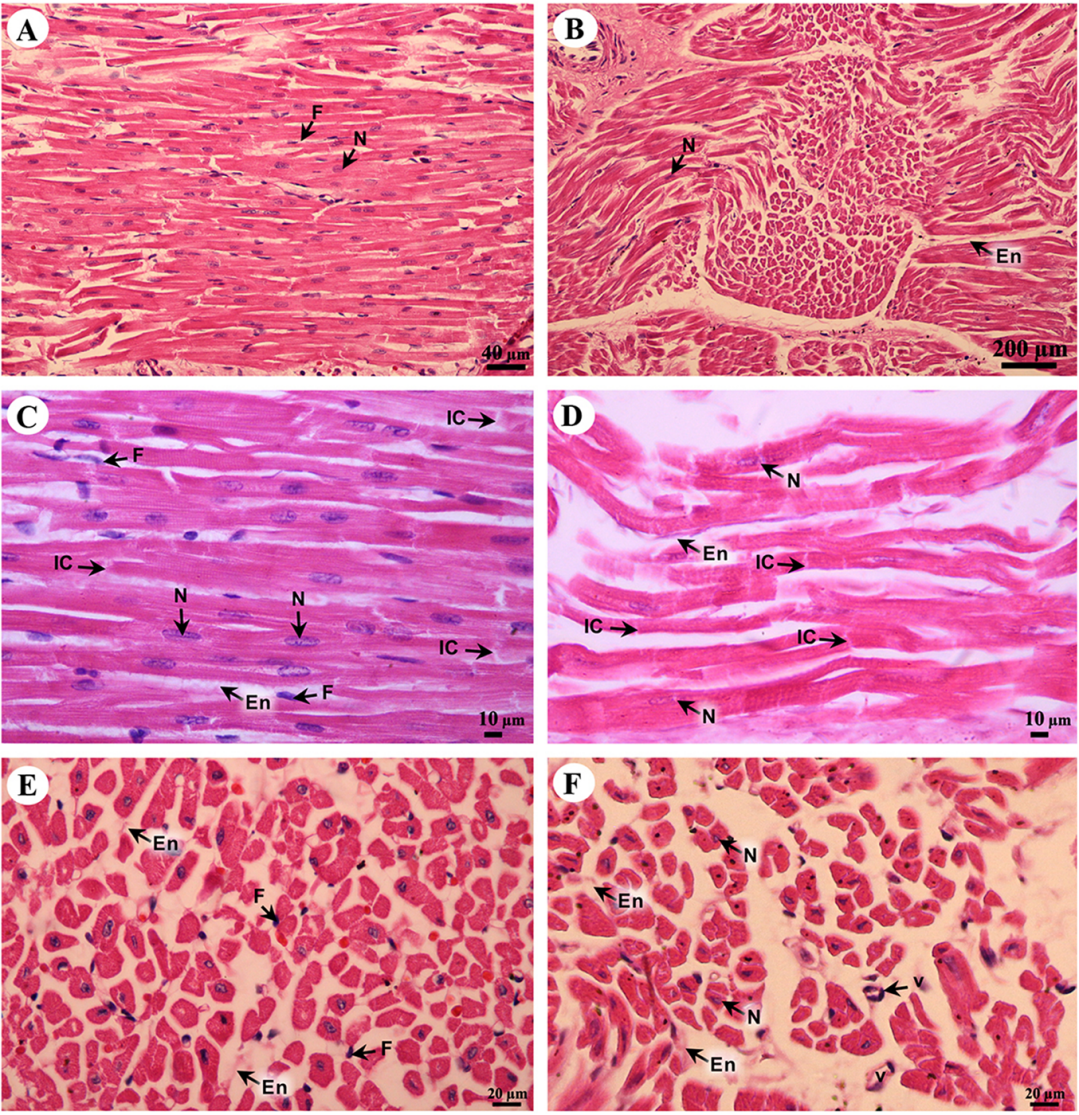
Low and high magnification of histological sections of cardiac muscle from right atrium (A, C, E) and right ventricle (B, D, F). Study sites: En, endomysium; F, fibroblast; IC, intercalated discs; N, nucleus; V, blood vessel. Hematoxylin and eosin staining.

Cardiac cells (cardiomyocytes) were separated by the endomysium, whereas nuclei and fibroblasts could be observed at low magnification (Figs. 8A and 8B). At high magnification, an oval-shaped nucleus was observed in the center of the cardiac cell, while fibroblasts were observed in the lateral border of the cell. Moreover, the junctions of cardiac cells, intercalated discs, were observed in a longitudinal section of cardiac muscle in both the right atrium and ventricle (Figs. 8C and 8D). In addition, high magnification of the cross-sectional area confirmed that cardiac cells were separated by the endomysium; the nucleus was in the center of the cardiac cell, whereas fibroblasts were on the lateral border of the cells (Figs. 8E and 8F). Moreover, at high magnification, myofilaments (indicated by red-stained pinpoints) were observed in the cross-sectional area of cardiac cells (Figs. 8E and 8F).

**Figure 9:**
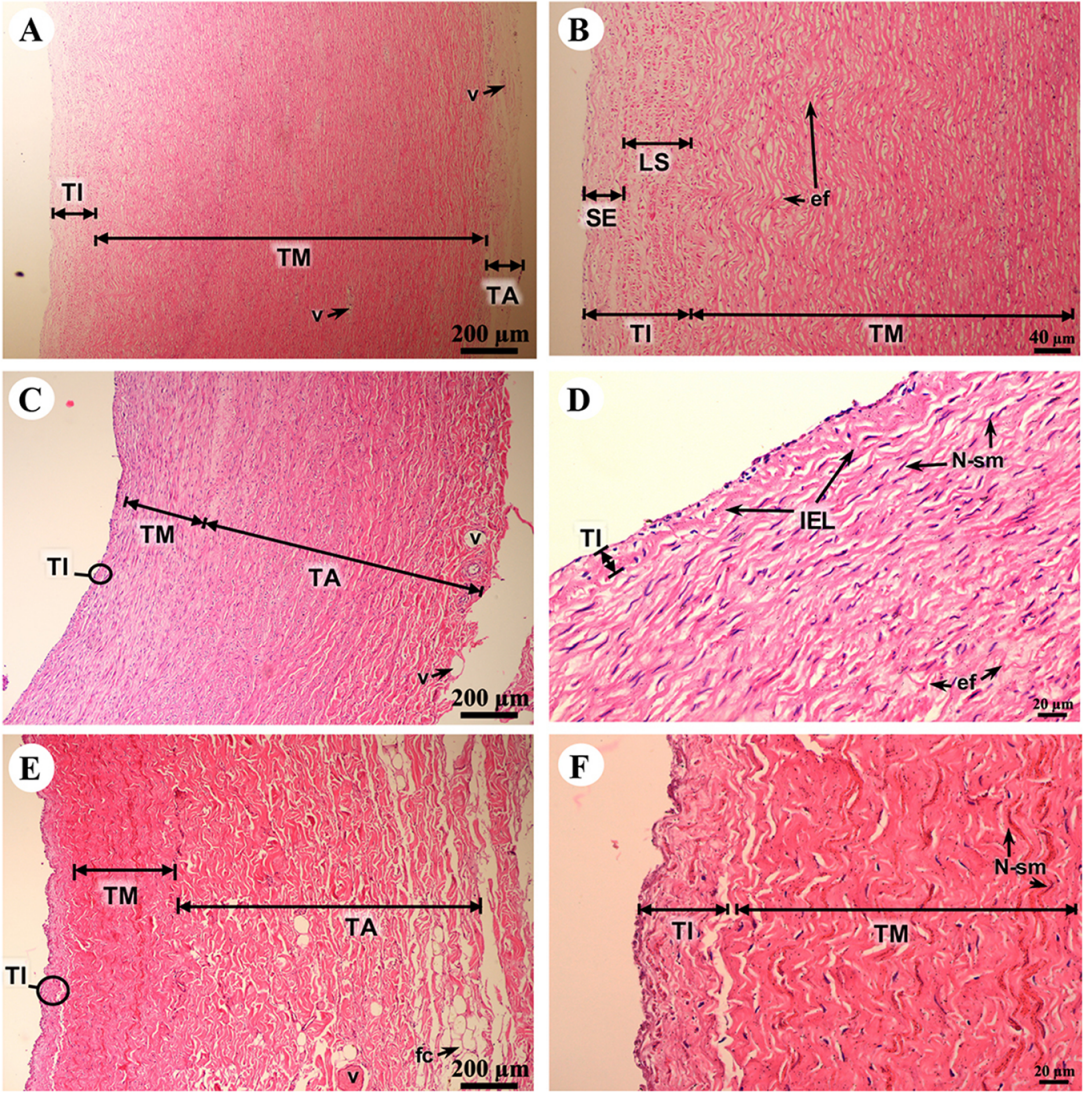
Low and high magnification of histological sections of the aorta (A, B), pulmonary artery (C, D) and caudal vena cava (E, F). Study sites: ef, elastic fiber; fc, fat cell; LS, longitudinal striated layer; IEL, internal elastic lamina; N-sm, nucleus of smooth muscle; SE, subendothelial layer; TA, tunica adventitia; TI, tunica intima; TM, tunica media; V, blood vessel. Hematoxylin and eosin staining.

The tunica intima, tunica media and tunica adventitia were found in all blood vessels. The aortic vessel, known as an elastic artery (Figs. 9A and 9B), demonstrated a thick tunica intima when compared to the pulmonary artery which is a muscular artery (Figs. 9C and 9D). In addition, in the tunica intima of the aorta subendothelial and longitudinal striated layers were observed, whereas in the muscular artery and the caudal vena cava these layers were not found (Figs. 9B, 9D and 9F). The tunica media, which consists of smooth muscle cells and elastic fibers, was found in all type of blood vessels (Figs. 9B, 9D and 9F); the aorta had the largest tunica media area when compared to small arteries and large veins (Figs. 9A, 9C and 9E). The outer part of the vessel is the tunica adventitia, which consists of small vessels and fat cells. In addition, the tunica adventitia in the elastic artery was found to have less area than in the muscular artery and large vein (Figs. 9A, 9C and 9E).

### Respiratory system

**Figure 10:**
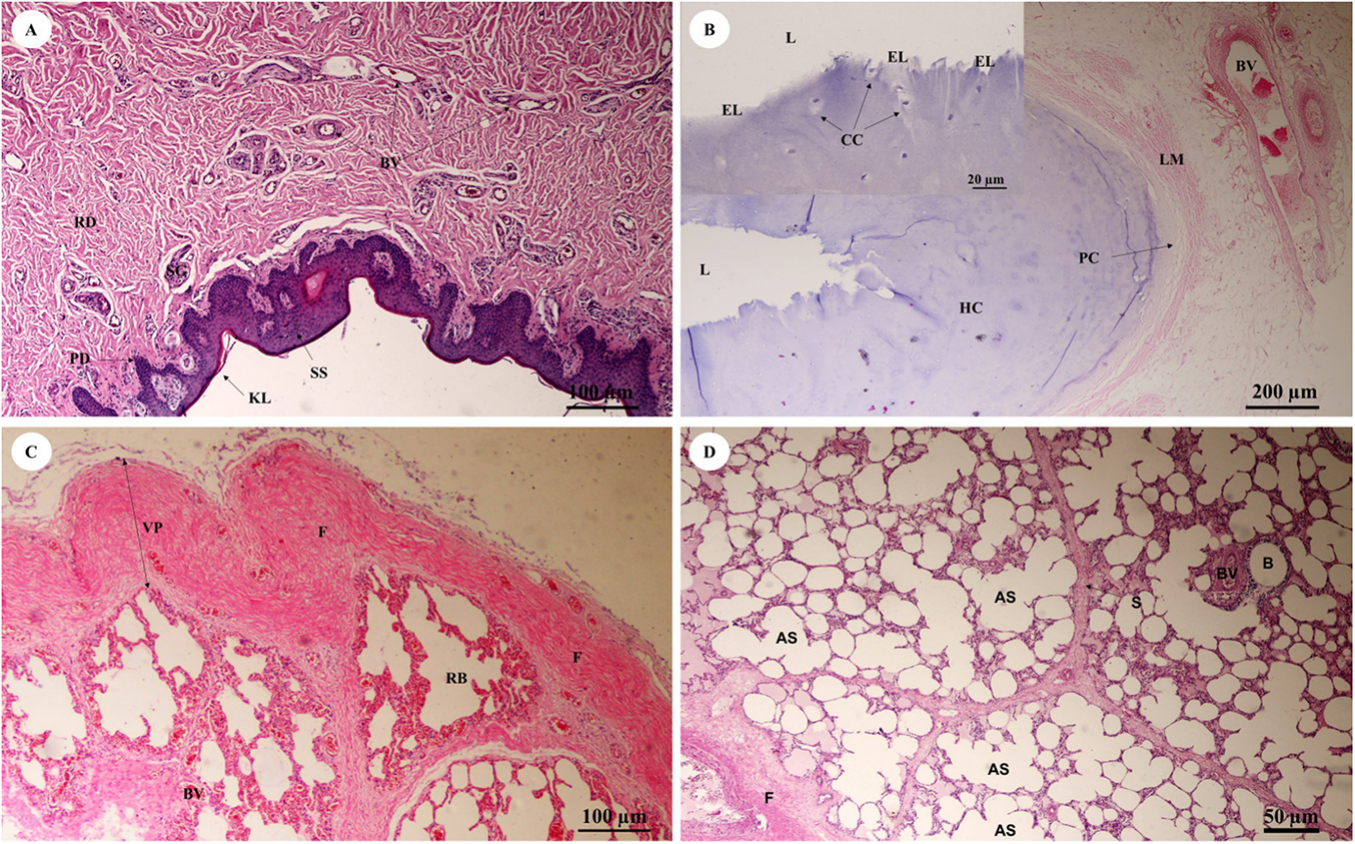
Low and high magnification of histological sections of the trunk (inner) (A), trachea (lower part) (B), pleura and intralung septa (C, D). Study sites: AS, alveolar sac; B, bronchiole; BV, blood vessel; CC, chondrocyte cells; EL, epithelial lining; F, fibrous supporting tissue; HC, hyaline cartilage; KL, keratinized layer; L, lumen; LM, longitudinal muscle; PC, perichondrium; PD, papillary dermis; RB, respiratory bronchiole; RD, reticular dermis; S, septum; SG, secretory gland; SS, stratified squamous epithelium; VP, visceral pleura. Hematoxylin and eosin staining.

The trunk (inner) (Fig. 10A) presented a structure similar to the skin epithelium: stratified squamous epithelium with a keratinized layer and dense collagenous fibrins in the dermis, but few sweat or sebaceous glands. In the trachea (lower part) (Fig. 10B), hyaline cartilage was distributed throughout, in lacunae with chondrocytes, longitudinal muscle or trachealis muscle around the trachea. No respiratory epithelial lining was found, which may be a result of improper preservation methods. Thickening of the visceral pleura (Fig. 10C) was observed, with fibrous supporting tissu. Intralung septa (Fig. 10D) separated lung parenchyma along with fibroelastic tissues.

**Figure 11:**
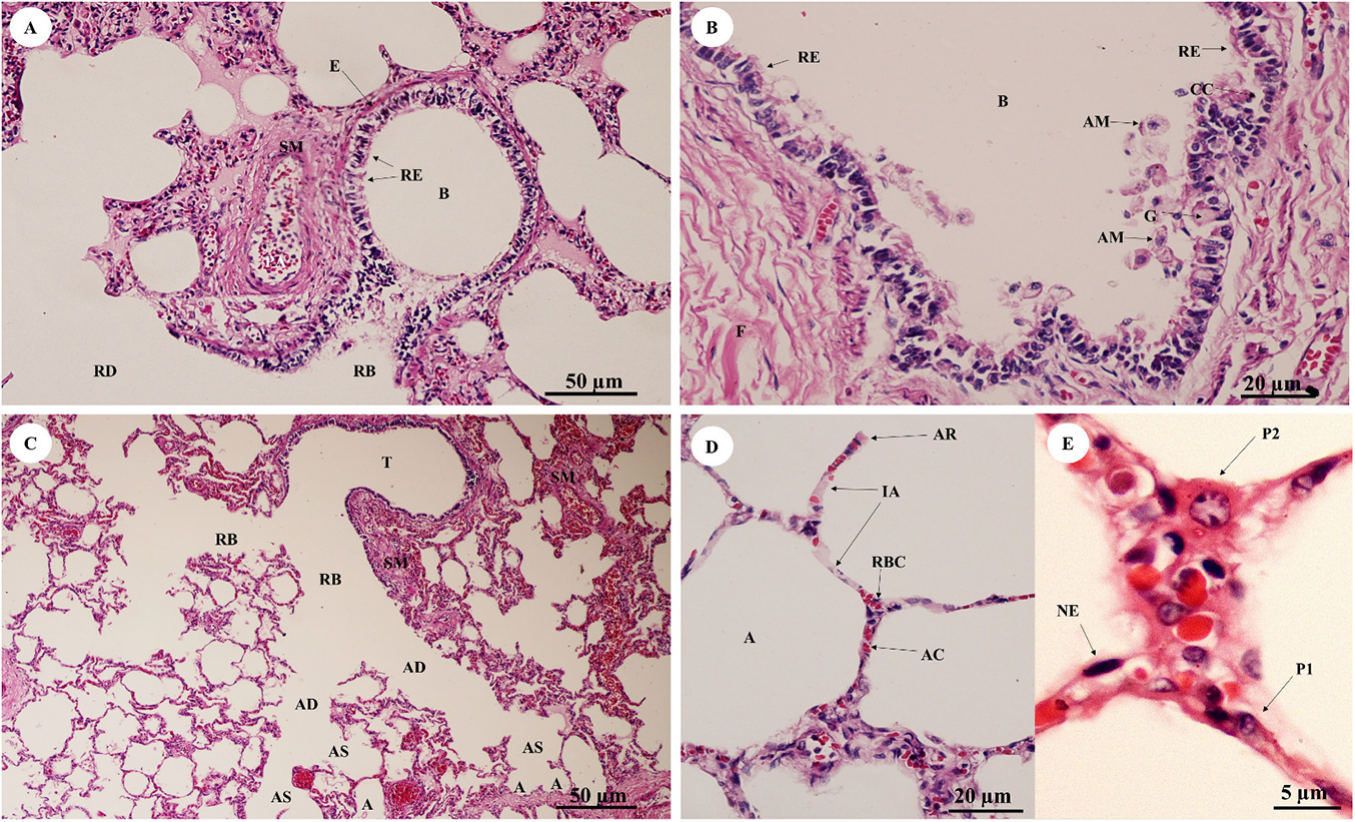
Low and high magnification of histological sections of bronchioles (A, B), respiratory tract (C), alveolar lining (D) and pneumocytes (E). Study sites: A, alveoli; AC, alveolar capillary; AD, alveolar duct; AM, alveolar macrophage; AR, alveolar ring; AS, alveolar sac; B, bronchiole; CC, Clara cell; E, elastic band; G, goblet cell; IA, interalveolar septum; NE, nucleus of endothelium; P1, pneumocyte type I; P2, pneumocyte type II; RB, respiratory bronchiole; RBC, red blood cell; RD, respiratory duct; RE, respiratory epithelium; SM, smooth muscle; T, terminal bronchiole. Hematoxylin and eosin staining.

In the bronchioles (Figs. 11A and 11B), the epithelial lining was composed of tall, pseudostratified columnar cells with cilia, goblet cells, alveolar macrophages and Clara cells (non-ciliated cuboidal epithelium), which have three primary functions: they produce pulmonary surfactant, act as stem cells, and contain enzymes for detoxifying noxious substances. There was thickening of fibroelastic tissues, forming an elastic band around the bronchioles, but few seromucinous glands in the submucosa and neither cartilage nor mucosa-associated lymphoid tissue (MALT) in the wall. In the respiratory tract (Fig. 11C), terminal bronchioles divided into respiratory bronchioles, which in turn divided into alveolar ducts, alveolar sacs and alveoli, respectively. Thickened smooth muscle, collagen and elastin fibers surrounded the terminal bronchioles and alveolar ducts. At higher magnification, the alveolar lining (Fig. 11D) showed type I pneumocytes (squamous cells) and type II pneumocytes (large and round-shaped) (Fig. 11E). The alveolar wall or interalveolar septum contained a dense network of alveolar capillaries filled with red blood cells. Small aggregations of smooth muscle cells, collagen and elastin fibers formed alveolar rings or knobs at the end of the alveolar wall.

### Gastrointestinal system

Gastrointestinal or digestive organs included tubular organs, i.e., esophagus, stomach (cardia, fundus, pylorus), small intestine (duodenum, jejunum, ileum) and large intestine (cecum, colon, rectum), and accessory organs, i.e., tongue, liver and pancreas. Histological examination of the tissues revealed that the tubular organs consisted of four layers. The layers of the gastrointestinal tract from the lumen to the external surface included the tunica mucosa, tunica submucosa, tunica muscularis and tunica serosa (tunica adventitia in the esophagus).

**Figure 12:**
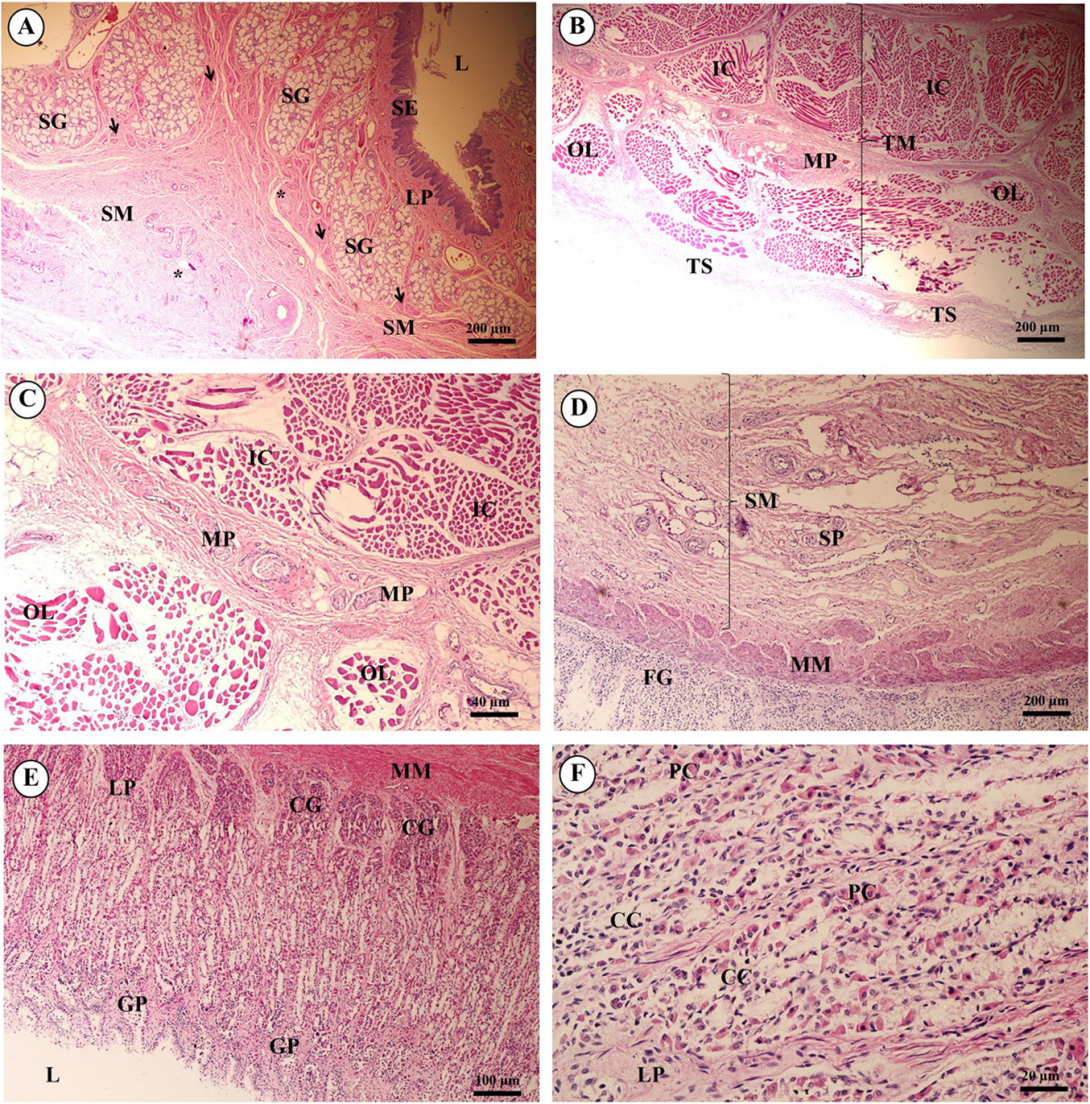
Light microscopy micrographs at different magnifications of the esophagus (A–C), cardiac, fundic and pyloric gland regions of the stomach (D–F). Study sites: Arrow, muscularis mucosae; Asterisk, submucosal nerve plexus; CG, cardiac gland; FG, fundic gland; GP, gastric pit; IC, inner circular muscle; L, lumen; LP, lamina propria; MP, myenteric nerve plexus; OL, outer longitudinal muscle; SE, surface epithelium; SG, submucosal mixed gland; Asteriskor SP, submucosal nerve plexus; SM, tunica submucosa; TM, tunica muscularis; TS, tunica serosa. Hematoxylin and eosin staining.

In the esophagus (Fig. 12), the non-keratinized stratified squamous epithelium of the tunica mucosa was markedly folded and had a slightly thick epithelium. The lamina propria consisted of an elaborate network of collagen fibers which contained tiny blood vessels. The connective tissue of the lamina propria was denser than that of the tunica submucosa. The muscularis mucosae or muscularis interna consisted of isolated bundles of smooth muscle in the submucosa above and between groups of submucosal glands. The loose connective tissue of the tunica submucosa contained numerous large blood vessels, the submucosal (Meissner’s) nerve plexus, and plentiful mixed seromucous glands with mucous glands predominating; several large ducts lined by stratified cuboidal epithelium opened into the luminal surface (Fig. 12A). In the tunica muscularis (or muscularis externa), inner circular and outer longitudinal layers were commonly seen, which were essentially separated by connective tissue and the myenteric (Auerbach’s) nerve plexus (Fig. 12B). Skeletal muscle formed the tunica muscularis, as confirmed by identification of cross-striations (Fig. 12C). The outer layer was the tunica adventitia, consisting of loose connective tissue containing blood vessels, lymphatic vessels and nerves.

As shown in Fig. 12, the proper gastric (cardiac, fundic and pyloric) region of the stomach was examined microanatomically, and the structure was found to be typical of monogastric domestic animals. In a number of sections the mucosal surface was autolytic, and it was not possible to discern the full extent of the mucosa or the gastric pits. The epithelial lining was tall simple columnar epithelium with oval or round nuclei basally located. The gastric pits, lined by simple columnar epithelium, were invaginations of the epithelial lining and were continuous with the gastric glands (Fig. 12D). The loose connective tissue of the lamina propria contained branched, straight tubular gastric glands in the fundic region, and branched, coiled tubular glands in the cardiac and pyloric regions. Chief cells, with basophilic cytoplasm, constituted the majority of cells; the parietal cells were large and the cytoplasm was eosinophilic (Fig. 12E). The muscularis mucosae were multiple indistinct discontinuous layers of smooth muscle with large amounts of interspersed collagen. The tunica submucosa of the stomach was a loose connective tissue consisting of collagen fibers with nerve plexuses, blood and lymphatic vessels, and other free cells. The tunica muscularis consisted of three layers of smooth muscle, the orientation of which was difficult to discern. The inner layers were oblique, and the middle circular and outer longitudinal layers in orientation of the tunica muscularis. A myenteric nerve plexus was located between the middle and outer layers. A serosal lining was seen in the tunica serosa and consisted of mesothelium overlying loose connective tissue (Fig. 12F).

**Figure 13:**
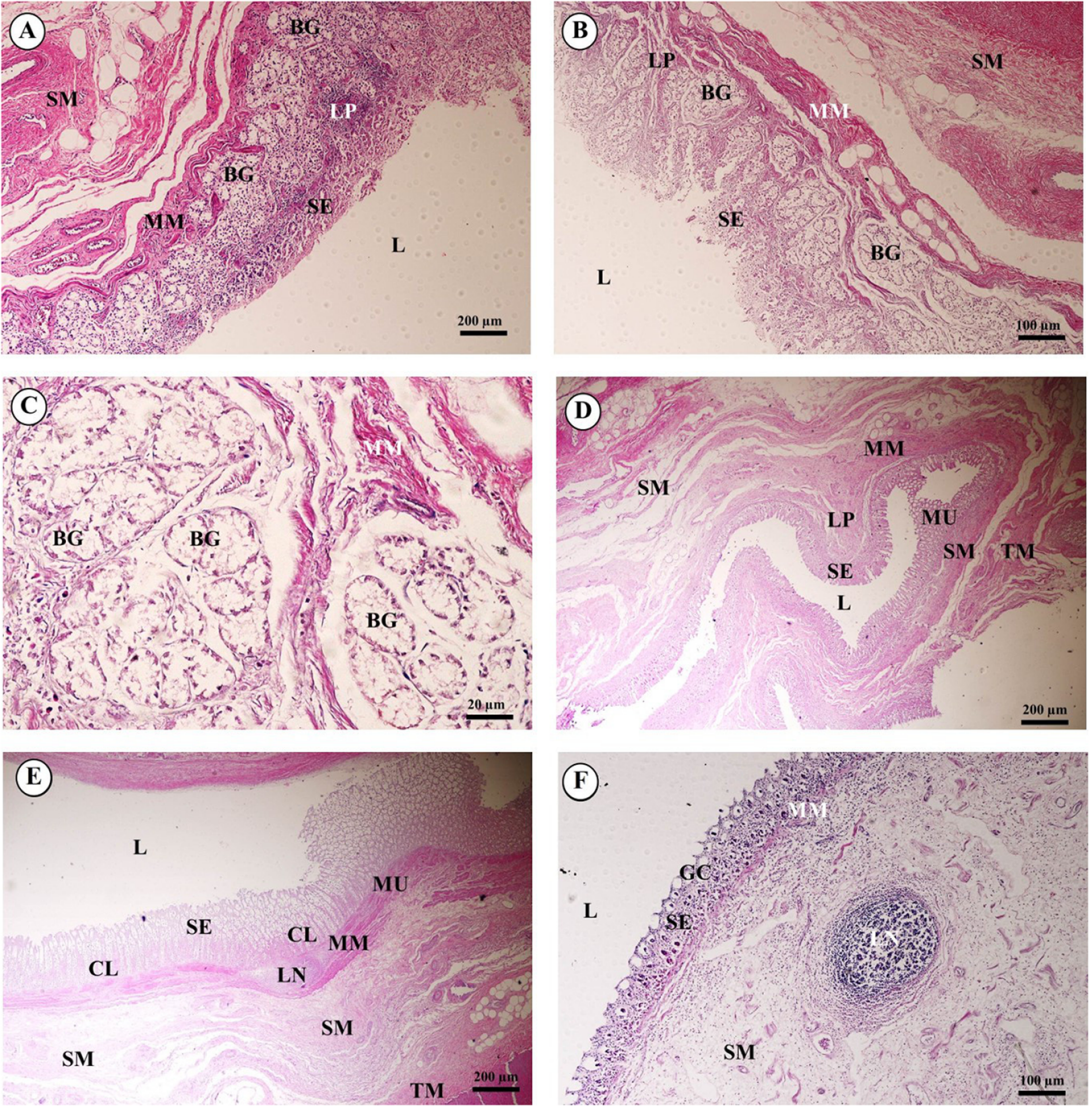
Light microscopy micrographs at different magnifications ofthe small intestine (duodenum, jejunum, ileum) (A–C) and large intestine (cecum, colon, rectum) (D–F). Study sites: BG, Brunner’s gland; CL, crypts of Lieberkühn; GC, goblet cell; L, lumen; LP, lamina propria; LN, lymphatic nodule; MM, muscularis mucosae; MU, tunica mucosa; SE, surface epithelium; SM, tunica submucosa; TM, tunica muscularis. Hematoxylin and eosin staining.

Three parts of the small intestine (duodenum, jejunum and ileum) were examined. Ciliated simple columnar intestinal epithelium with goblet cells lined the tunica mucosa. The villi were long and irregular in outline, and simple tubular glands (crypts of Lieberkühn) opened between the bases of the villi and extended to the muscularis mucosae (Fig. 13A). The mucosal surface was autolytic, while Paneth cells were not apparent and mucosal folds were not prominent. The prominence of the muscularis mucosae varied from a thin area of indistinct multiple layers to more extensive bundles of smooth muscle in poorly defined layers (Fig. 13B). The submucosa was generally thick, with large amounts of collagen, large blood vessels, nerves, and a prominent submucosal nerve plexus. Brunner’s glands (compound tubular submucosal glands) were prominent within the tunica submucosa of the duodenum (Fig. 13C). The tunica muscularis consisted of an inner circular layer and an outer longitudinal layer of smooth muscle between which the myenteric nerve plexus was prominent. The tunica serosa consisted of a thin layer of loose connective tissue covered by mesothelium.

The mucosa of the large intestine had prominent mucosal folds. The lumen was lined by a simple columnar epithelium with a brush border, and numerous long, straight tubular glands (crypts of Lieberkühn). Plentiful goblet cells were observed, particularly in the crypts, and were more numerous in the large intestine than in the small intestine (Fig. 13D). The lamina propria was relatively cellular, with plentiful lymphocytes and plasma cells and smaller numbers of polymorphonuclear cells. The muscularis mucosae was generally prominent and consisted of several indistinct discontinuous layers with no obvious organization; the smooth muscle of this layer was admixed with the dense connective tissue of the submucosa. Lymphoid follicles extended from the lamina propria to the tunica submucosa, and heavy infiltration of the lamina propria with lymphoid cells was observed. The tunica submucosa was a loose connective tissue, with collagen, large blood vessels, nerves, and a prominent submucosal nerve plexus. The tunica muscularis was thick in most of the sections examined; the inner circular layer of smooth muscle was thicker than the outer longitudinal layer, and between the two layers a prominent myenteric nerve plexus and blood vessels were seen (Figs. 13E and 13F). The connective tissue of the tunica serosa extended into the outer longitudinal layer of the tunica muscularis, forming indentations within this layer.

**Figure 14:**
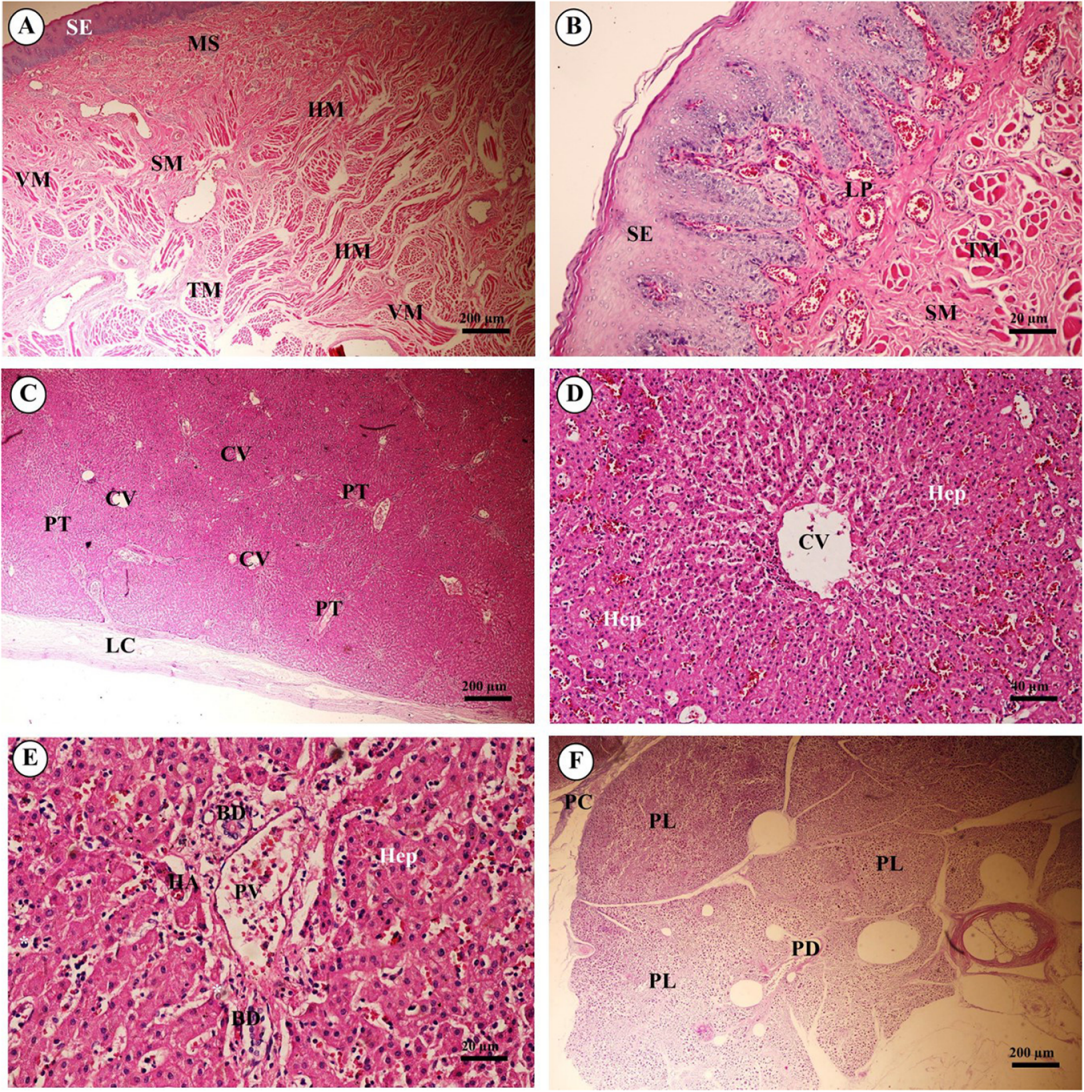
Light microscopy micrographs at different magnifications ofthe tongue (A, B), liver (C–E) and pancreas (F). Study sites: BD, bile ductule; CV, central vein; HA, hepatic artery; Hep, hepatocytes;HM, horizontal muscle; LC, liver capsule; LP, lamina propria; MS, minor salivary gland; PC, pancreatic capsule; PD, pancreatic duct; PL, pancreatic lobule; PT, portal triads; PV, portal vein; SE, surface epithelium; SM, tunica submucosa; TM, transverse muscle. Hematoxylin and eosin staining.

The tongue, an accessory digestive organ, was lined by a mucous membrane consisting of non-keratinized stratified squamous epithelium and an underlying connective tissue layer. The lamina propria merged with the tunica submucosa, forming a propria-submucosa. The surface was kept moist with mucus produced by numerous minor salivary glands. No papillae were observed on the dorsal surface of the tongue. The body of the tongue was composed of intertwined bundles of skeletal muscle: transverse, longitudinal and horizontal muscles. The dense lamina propria was continuous with the connective tissue of the muscle, tightly binding the mucous membrane to the muscle (Figs. 14A and 14B).

The liver was covered by the serosa (visceral peritoneum), or liver capsule. Smooth muscle, elastic and collagen fibers formed the dense connective tissue of the liver capsule (Fig. 14C). Interlobular connective tissue had variable amounts of collagen, and smooth muscles were seen around the central veins (Fig. 14D). The portal areas, or portal triads, were typical structures, consisting of one or more branches of the hepatic artery, portal vein and bile ductule within a connective tissue framework. A smooth muscle sphincter was not observed around the portal vessels, and the amount of connective tissue within the portal areas was variable. The bile ductule within the portal areas was lined by a simple cuboidal epithelium, and larger bile ducts were lined by a simple columnar epithelium. The hepatocytes were arranged in rows, or laminae, of single-layered cells, separated by sinusoids lined by endothelial cells and Kupffer cells (hepatic macrophages). Hepatocytes had a centrally located nucleus with prominent nucleoli; the cytoplasm appeared granular and contained vacuoles (lipid droplets) (Fig. 14E).

Pancreas tissues were autolytic, and exocrine and endocrine parts could not be identified. The pancreas was surrounded by a thin capsule of connective tissue. Septa from the capsule extended into the parenchyma, dividing it into lobules; within the interlobular connective tissue, nerve fibers and ganglia, blood vessels, and interlobular ducts lined by simple cuboidal to low columnar epithelium, or simple columnar epithelium in the larger ducts, were seen. Within the exocrine component of the pancreas, the tubuloacinar secretory unit was composed of glandular epithelial cells arranged as acini surrounding a small lumen. The cells were pyramidal in shape, had a basal spherical nucleus surrounded by basophilic cytoplasm, and the apical cytoplasm of these cells was filled with eosinophilic granular material (zymogen) (Fig. 14F).

### Urinary system

**Figure 15:**
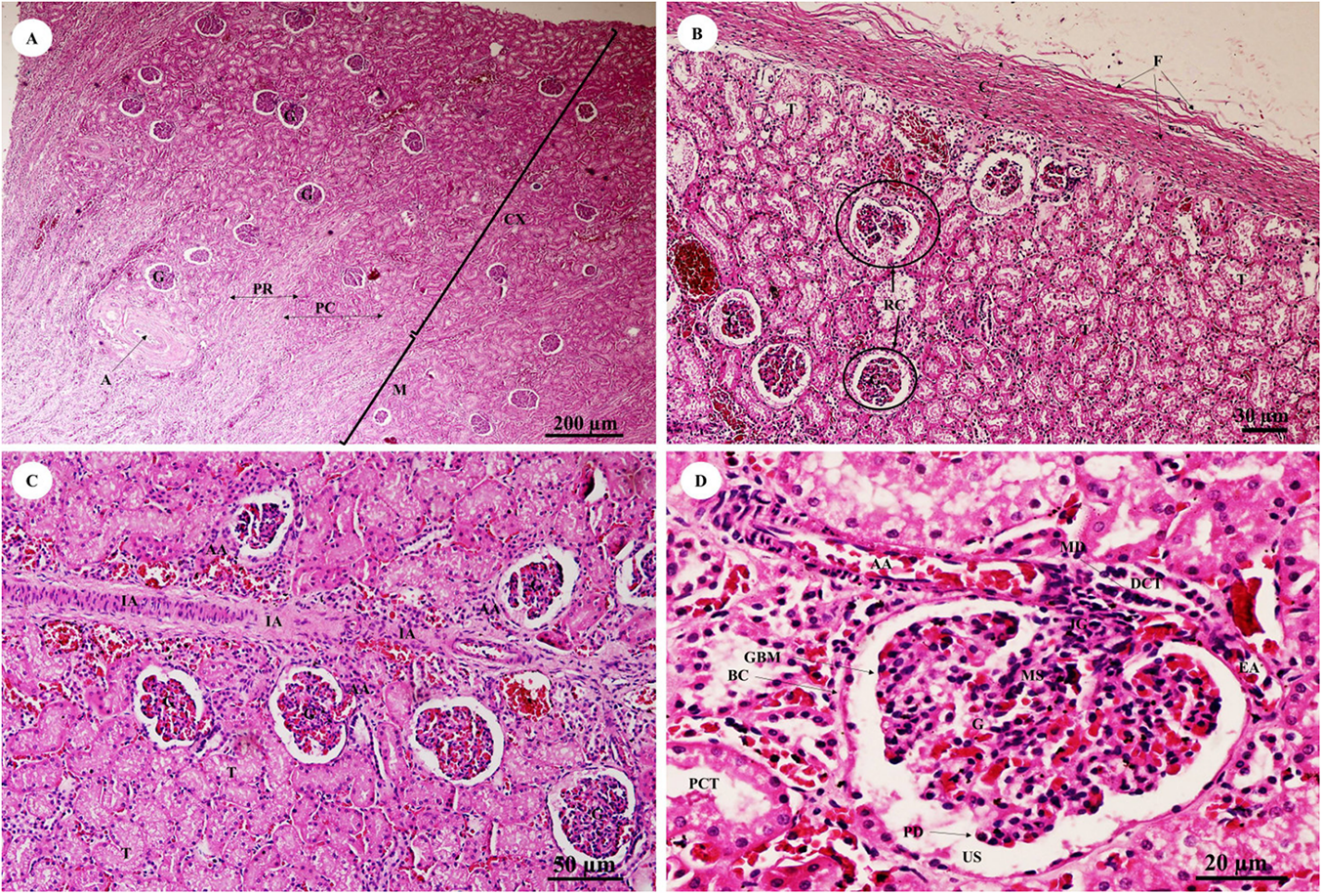
Low and high magnification of histological sections of the renal cortex and medulla and renal capsule (A, B), renal vessels (C) and glomerulus complex (D). Study sites: A, arcuate artery; AA, Afferent arteriole; BC, Bowman’s capsule (parietal epithelial cell); C, renal capsule; CX, renal cortex; DCT, distal convoluted tubule; EA, efferent arteriole; F, fibrous connective tissue; G, glomerulus; GBM, glomerular basement membrane; IA, intralobular artery; JG, juxtaglomerular cell; M, renal medulla; MD, macula densa; MS, mesangial cell; PC, pars convoluta; PCT, proximal convoluted tubule; PD, podocyte cell; PR, pars radiata (medullary ray); RC, renal corpuscle; T, tubule; US, urinary space. Hematoxylin and eosin staining.

The kidney was divided into two parts, the renal cortex and renal medulla (Fig. 15A), with numerous glomeruli (condensed capillaries) in the cortex and a few in the medulla. Pars radiata or medullary rays consisted of straight tubules, while the pars convoluta consisted of convoluted tubules. The smallest function unit of the kidney is called the nephron, which is composed of a glomerulus, Bowman’s capsule, proximal convoluted tubule, Henle’s loop, distal convoluted tubule and collecting tubules. The arcuate artery is located between the cortex and medulla, or corticomedullary junction. The renal capsule (Fig. 15B), a tough fibrous layer of collagen and elastic tissues, encapsulated the kidney. Renal vessels (Fig. 15C) included the intralobular artery, which branches from the arcuate artery and provides blood supply to each glomerulus via afferent arterioles. At high magnification (Fig. 15D), a cross section of the glomerulus complex showed juxtaglomerular cells lining the afferent arteriole wall, mesangial cells (forming the central region of the glomerulus), podocytes (foot cells) are finger-like processes along with glomerular capillaries. The macula densa, an area of densely packed columnar cells, lined the wall of the distal convoluted tubule where it attached to the glomerulus. The glomerulus has two layers: the visceral layer, or glomerular basement membrane, and the parietal layer, or Bowman’s capsule, which is simple squamous epithelium. Urinary space is the space between these layers into which ultrafiltrated fluid enters.

**Figure 16:**
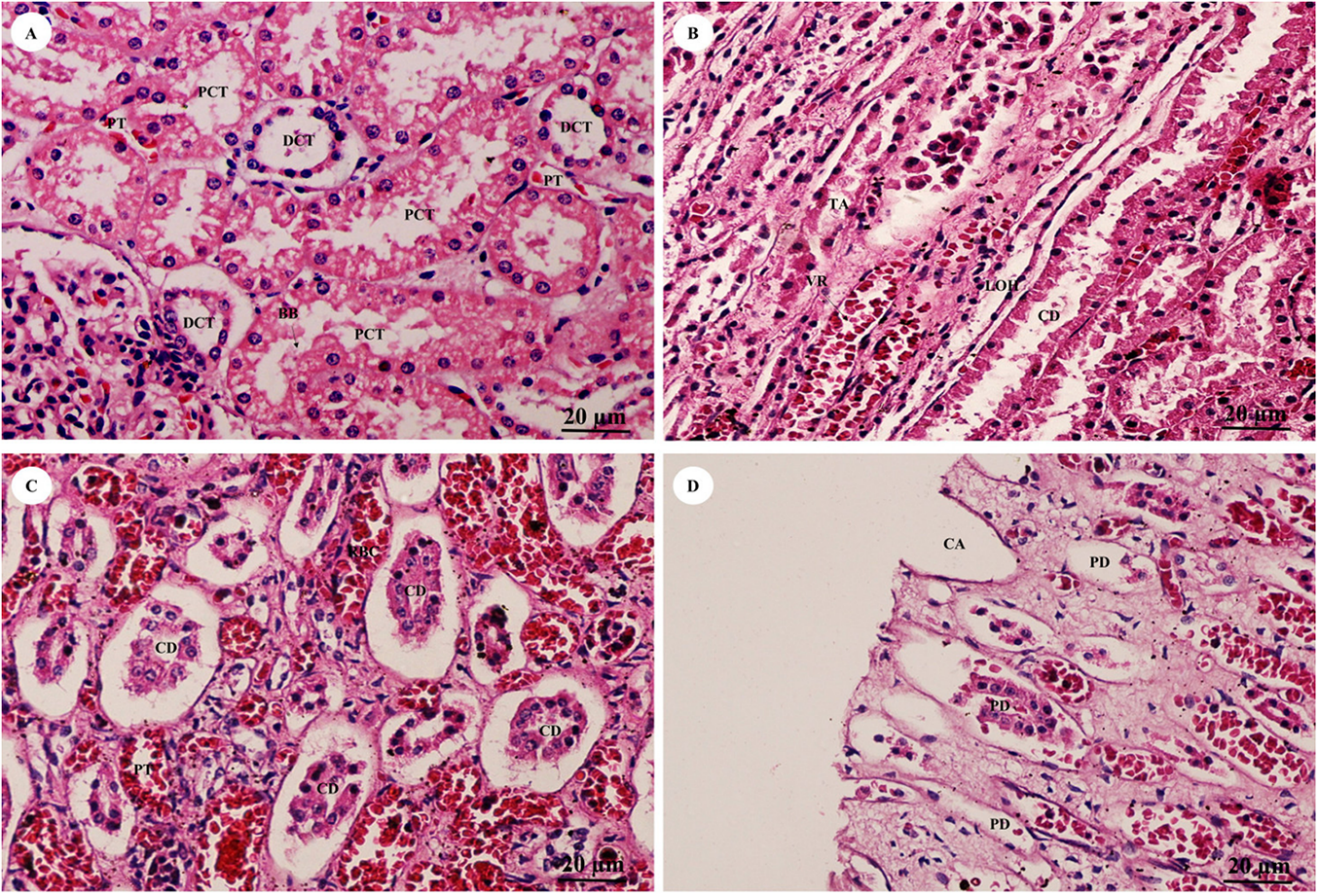
High magnification of histological sections of pars convoluta (A), pars radiata (B), collecting duct (C) and renal papilla (D). Study sites: BB, brush border; CA, cribriform area; CD, collecting duct; DCT, distal convoluted tubule; LOH, thin segment Henle’s loop, PCT, proximal convoluted tubule; PD, papillary duct; PT, peritubular capillary; RBC, red blood cell; TA, thick ascending Henle’s loop; VR, vasa recta. Hematoxylin and eosin staining.

The pars convoluta (Fig. 16A) is the convoluted part of the renal cortex, and consists of proximal and distal convoluted tubules. The proximal convoluted tubule is simple cuboidal epithelium with a brush border or microvilli, exhibiting dark staining and a round-shaped nucleus; while the distal convoluted tubule is simple cuboidal epithelium with no brush border, a large lumen and lighter staining than proximal tubule cells. Pars radiata (Fig. 16B) or medullary rays are straight portions of the renal medulla, consisting of straight renal tubules distributed throughout thick ascending and descending limbs (simple cuboidal epithelium) and thin segmented Henle’s loops (simple squamous epithelium), along with peritubular capillaries or vasa recta. Collecting ducts (Fig. 16C) are simple columnar epithelium. Renal papilla (Fig. 16D) presented collecting ducts which collect ultrafiltrated fluid from distal tubules and drain into the papillary duct or duct of Bellini (high simple columnar epithelium, light staining, clear border) opening into the cribriform area and the renal pelvis.

### Reproductive system

**Figure 17:**
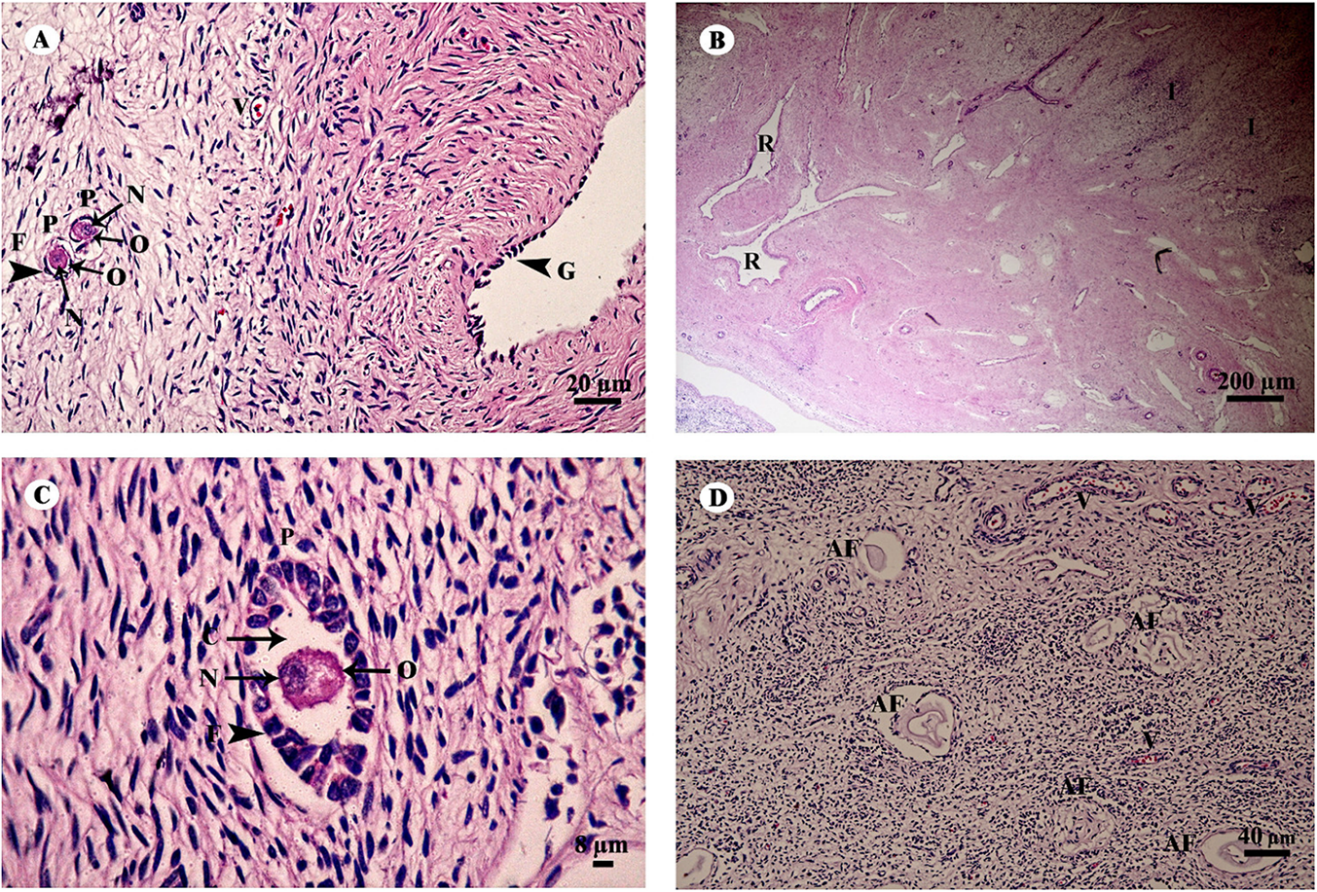
Low and high magnification of histological sections of the ovary (A–D). Study sites: AF, atretic follicle; C, cytoplasm; F, granulosa cell; G, germinal epithelium; I, interstitial cell; N, nucleus; O, oocyte; P, primary oocyte; R, rete ovarii; V, blood vessel. Hematoxylin and eosin staining.

The ovary was divided into two components, an outer cortex (Figs. 17A and 17C) and an inner medulla (Fig. 17B). The cortex was covered by a single layer of cuboidal cells called the germinal epithelium (Fig. 17A). The surface epithelium was located on a basement membrane, and beneath the germinal epithelium was a layer of dense connective tissue, the tunica albuginea, forming a narrow layer of cells running parallel to and just beneath the ovarian epithelium (Fig. 17A). The cortical stroma included ovarian follicles in various stages of development extending deep into the tunica albuginea (Fig. 17A). The medulla was composed of vascularized loose connective tissue. Furthermore, this component contained an accumulation of darkly stained interstitial cells, blood vessels, and rete ovarii were also observed within the medulla (Fig. 17B). Growing small follicles up to the stage of small antral follicles were present, comprised of an oocyte surrounded by a single layer of granulosa cells of varying thickness. The oocyte contained pale eosinophilic cytoplasm and a dark-staining nucleus (Figs. 17A and 17C). Primordial follicles are oocytes surrounded by a single layer of squamous granulosa cells. A few primordial follicles were found in this tissue. However, early primary oocytes surrounded by a single layer of granulosa cells, of which at least one was cuboidal, were found abundantly in this specimen (Figs. 17A and 17C). Development of secondary follicles or Graafian follicles was not found in this elephant. The degenerative regression of the follicles, called follicular atresia, is characterized by an irregular outline of the follicle and separation of the granulosa cells (Fig. 17D).

**Figure 18:**
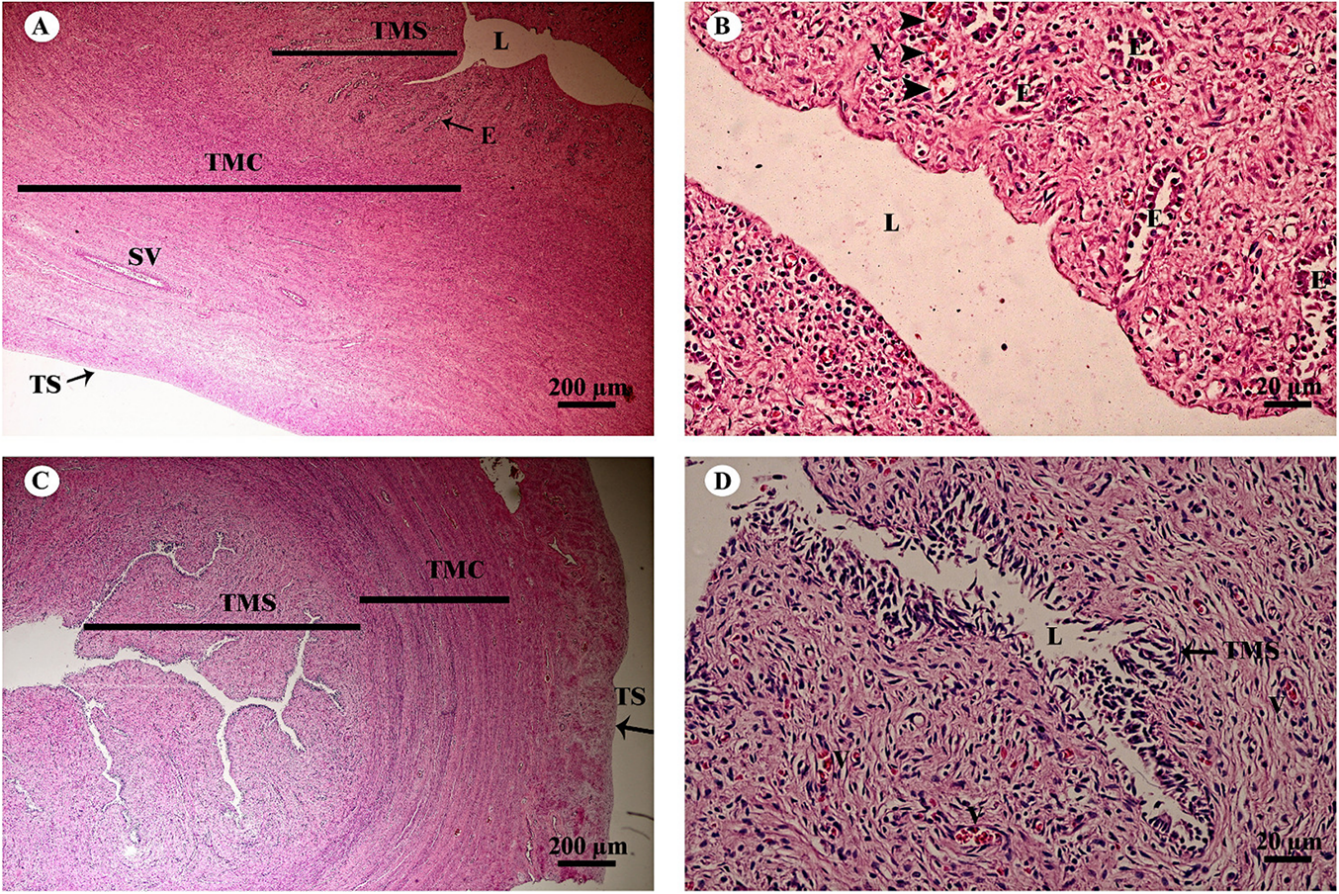
Low and high magnification of histological sections of the juvenile reproductively inactive uterine horn (A, B) and uterus (C, D). Study sites: E, endometrial gland or uterine gland; L, lumen; SV, stratum vasculare; TMC, tunica muscularis (muscularis); TMS, tunica mucosa (endometrium); TS, tunica serosa (perimetrium or serosa). Hematoxylin and eosin staining.

The uterus in elephants is bicornuate, with right and left horns, a body and a neck (cervix) (Fig. 18). The uterine wall in the uterine horns and body has three layers: an innermost endometrium (tunica mucosa), underlying middle myometrium (tunica muscularis) and an outermost perimetrium (tunica serosa) (Figs. 18A and 18C). The surface of the endometrial lining consisted of pseudostratified epithelium, connective tissue with simple tubular glands (Figs. 18B and 18D); uterine glands that open into the lumen of the uterus are coiled (Figs. 18B and 18D). The endometrium revealed irregularly raised longitudinal uterine folds and ridges which formed the star-shaped uterine lumen. A thick layer of myometrium surrounded this endometrial tissue, which was composed of a deep inner layer of circular smooth muscle and an outer layer of longitudinal smooth muscle (Figs. 18A and 18C). The stratum vasculare, a layer of connective tissue carrying the blood vessels to the uterus, divided the circular muscle into inner and outer layers (Fig. 18A). The perimetrium was the outermost loose connective tissue layer, or serosa, of the uterus (Figs. 18A and 18C).

### Lymphatic system

**Figure 19:**
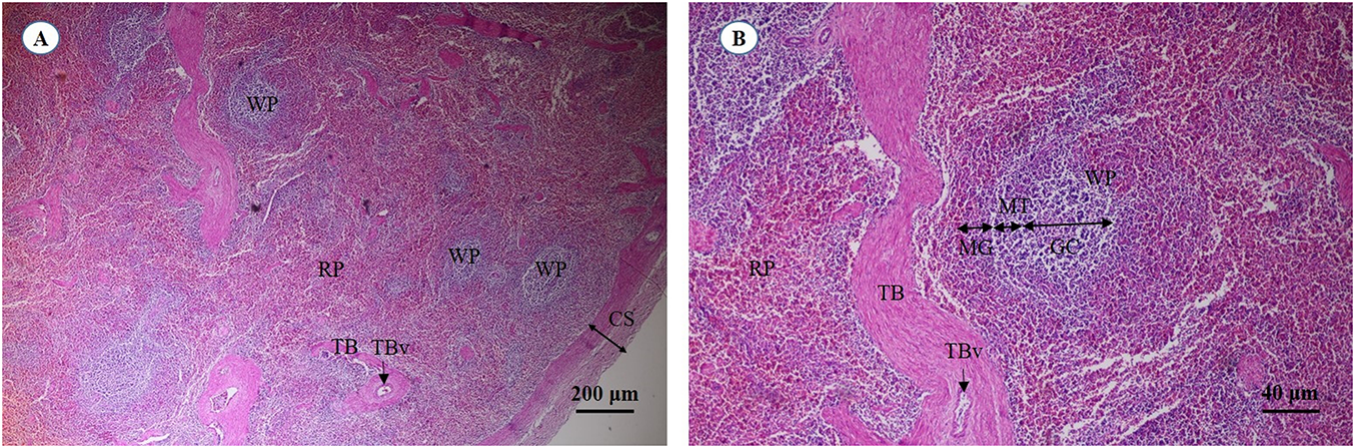
Light micrographs at different magnifications of the spleen (A, B). Study sites: CS, capsule; GC, germinal center; MG, marginal zone; MT, mantle zone; RP, red pulp; TB, trabecula; TBv, trabecular vessel; WP, white pulp. Hematoxylin and eosin staining.

A cross section of the spleen showed that lymphatic tissue was enclosed by dense connective tissue. The splenic trabeculae extended inward from the capsule, through which trabecular vessel. The splenic parenchyma was composed of two functionally and morphologically distinct compartments, red pulp and white pulp (Fig. 19A). The red pulp comprised a concentric meshwork of reticular tissue and splenic sinusoids where red blood cells accumulate. The white pulp was subdivided into the periarterial lymphatic sheath, lymphatic nodules, and the marginal zone. The compartment of splenic nodules predominantly contained B-lymphocytes and T-lymphocytes, whereas the marginal zone consisted of macrophages, dendritic cells and lymphocytes (Fig. 19B).

## Discussion

This is the first report on the histological structure of 24 organs from a juvenile Asian elephant. In this regard, the present findings are important and provide valuable information. In future studies, our group will investigate the microanatomy of the same organs in an adult Asian elephant.

### Integument system

The histological findings of Asian elephant skin are similar to those of African elephants. In addition, the absence of both arrector pili muscles and sebaceous glands in the elephants has been reported (Spearman, 1970). The primary function of the pilosebaceous unit is production and secretion of sebum for covering the hair and skin (Zouboulis et al., 2009). The dryness skin of elephants, being pachyderms, is the result of the absence of pilosebaceous units. Moreover, no sudoriferous glands occur in *Elephas maximus* (Spearman, 1970). The common hippopotamus (*Hippopotamus amphibius*) also lacks dermal skin glands in the corium tissue (Allbrook, 1962). However, in the histological skin structure of the black rhinoceros (*Diceros bicornis*) and white rhinoceros (*Ceratotherium simum*), numerous large, simple coiled tubular sudoriferous glands are found in the dermoepidermal junction (Plochocki et al., 2017). The role of sudoriferous glands in body temperature regulation is well documented (Fuller et al., 2016). Therefore, the mechanism of heat transfer in elephants involves behavioral developments, including ear flapping, dust bathing, mud spraying and evaporative breathing (Rees, 2002). Since the elephant skin is the largest organ, future studies will have to clarify the different histological structures in various areas.

### Nervous system

The cerebral cortex of the Asian elephant can be divided into six layers, resembling those of the African elephant (Jacobs et al., 2011), manatee and giraffe (Jacobs et al., 2015, 2016; Reyes et al., 2016, 2015). Cerebellar cortex layers and neuronal cytoarchitecture of the Asian elephant are identical to those of afrotherians (African elephant, Florida manatee), carnivores (Siberian tiger, clouded leopard), cetartiodactyls (humpback whale, giraffe) and primates (human, common chimpanzee) (Jacobs et al., 2014; Maseko et al., 2013). Interestingly, the sciatic nerve of the Asian elephant contains numerous fascicles and very thick epineurium, in contrast to that of humans, dogs and chickens. To elucidate the cytoarchitecture of the different nervous tissues, specialized nervous tissue staining techniques and qualitative/quantitative methods should be employed.

### Skeletal system

The hyaline cartilage microstructure of juvenile Asian elephants was similar to that of juvenile and adult African elephants and other species (Egger et al., 2008). Plexiform bone is characteristic of cortical bone in large and/or fast-growing animals, such as horses, pigs, buffaloes, cows, goats and dogs, and occurs with less frequency in primates, including humans and monkeys (Hillier & Bell, 2007; Martiniaková et al., 2006; Mori et al., 2003; Nganvongpanit et al., 2015). In some species, like dogs, sheep and deer, plexiform bone is found only in immature animals (Hillier & Bell, 2007; Nganvongpanit et al., 2017a). Humeral bone of juvenile Asian elephants in our study did not contain plexiform bone, but this structure was found in flat bone taken from parietal bone. A previous study on neonatal and young juvenile African and Asian elephants reported the presence of plexiform bone in the femur and tibia (Curtin et al., 2012). It is possible that plexiform bone in long bone is present in neonatal and young juvenile elephants and develops into cortical bone very rapidly to increase bone strength during maturation. However, flat bone develops more slowly than long bone, so we found both woven and plexiform bone in the 2-year-old elephant. Double-zoned osteons, which have been reported in the adult Asian elephant (Nganvongpanit et al., 2017b), were not present in the juvenile elephant.

### Muscular system

The histological of striated muscle taken from a juvenile Asian elephant was similar to human and other animal models, including those of zebrafish, mice, dogs, cats (Aughey & Frye, 2001b; Lawlor et al., 2016), pigs and horses (Bacha & Bacha, 2000), as well as a previous study on woolly mammoths (*Mammuthus primigenius*) (Papageorgopoulou, Link & Ruhli, 2015). Hematoxylin and eosin staining demonstrated that muscle fibers in the cross-sectional area were equally distributed, with no difference in size, whereas in the juvenile Asian elephant there was increased space between muscle fibers. This was consistent with a previous study on Zimbabwean elephants (*L. africana*) with flaccid trunk paralysis; the animals exhibited increased space between muscle fibers and various sizes of muscle fibers in the cross-sectional area (Kock et al., 1994). These findings could be due to muscular atrophy (Aughey & Frye, 2001b), myotubular impairment (Lawlor et al., 2016) or nutritional deficiencies and toxicosis (Kock et al., 1994). In addition, although in this case the tissue was collected within 12 h, the timing of tissue collection is of critical importance and might affect the microanatomical structure. A previous study reported that enzymatic activity fluctuates greatly in the first 48 h, depending on storage temperature (Moore et al., 2015).

### Cardiovascular system

In this study, we found that the histology of cardiac muscle taken from a juvenile Asian elephant was similar to human and other animal models (Aughey & Frye, 2001b; Bacha & Bacha, 2000; Lawlor et al., 2016), but mostly similar to the horse which is another large mammal (Aughey & Frye, 2001b; Bacha & Bacha, 2000). Interestingly, we found that the right ventricle of the elephant contained many groups of muscle fascicles, the same as in the horse, whereas other species do not (Aughey & Frye, 2001b; Bacha & Bacha, 2000). This finding might be related to cardiac muscle mass. Hematoxylin and eosin staining demonstrated that one or two oval-shaped nuclei were located in the center of the cardiac cell; intercalated discs were found, similar to other species. Space between cardiac muscle fibers and decomposition of intercalated discs in these juvenile Asian elephants could be due to muscular atrophy (Aughey & Frye, 2001b), myotubular impairment (Lawlor et al., 2016), nutritional deficiencies and/or toxicosis (Kock et al., 1994), or the timing of tissue collection (Kachaeva & Shenkman, 2012).

The microstructure of the aorta, an elastic artery, demonstrated that the tunica intima, tunica media and tunica adventitia were similar to the horse (Aughey & Frye, 2001b) and pig (Bacha & Bacha, 2000). In addition, hematoxylin and eosin staining of the aorta revealed a high proportion of wavy elastic fibers in the tunica media, the same as in the horse, which allows the vessels to dilate and recoil back to normal (Aughey & Frye, 2001b). Previous studies in the pig, horse and dog did not find layer separation in the aortic tunica intima (Aughey & Frye, 2001b; Bacha & Bacha, 2000; Orsi et al., 2015). Interestingly, we found that aortic tissue from the juvenile Asian elephant had two layers (subendothelial and longitudinal striated layer) of the tunica intima, a finding consistent with the aortic tunica intima in humans (Shu-Xin, 1999). In the medium-sized muscular artery, a previous study demonstrated a reduction in elastic fiber (tunica adventitia) and an increase in smooth muscle (tunica media) (Aughey & Frye, 2001b); however, our findings showed that the pulmonary artery had a high proportion of elastic fibers (tunica adventitia), whereas the proportion of smooth muscle (tunica media) was decreased. This discrepancy may be due to the size of the muscular artery, which affects the proportion of the tunica media and tunica adventitia (Bacha & Bacha, 2000). The large vein (caudal vena cava) taken from a juvenile Asian elephant in this study demonstrated that the microstructure was similar to that of the dog, which has a higher proportion of the tunica adventitia compared to the tunica media (Bacha & Bacha, 2000). Moreover, the tunica adventitia of the juvenile Asian elephant was composed of elastic fibers, small arteries and veins in the walls of the larger blood vessels, the same as in dogs (Aughey & Frye, 2001b; Bacha & Bacha, 2000).

### Respiratory system

The elephant’s trunk is a nostril, a long air-conducting passage through to the lung. This long nasal tube may have evolved for breathing while swimming or walking across a river (West, 2001, 2002). The histology of the trunk (inner) is like that of the upper respiratory tract, stratified squamous epithelium with a keratinized layer and dense collagenous fibers in the dermis, similar to normal skin but with a few sweat or sebaceous gland in the submucosa. The trachea is at the lower part of the trunk. The elephant’s trachea is short, about 12 inches in length, and is composed of 25 cartilage rings which are almost completely hyaline cartilage (Brown et al., 1997). The thin trachealis muscle is unlike that of the cat, which has a thick muscle (Stirling, 1883). No serous/mucinous glands or lymphoid cells were found in the submucosa, similar to the horse and albino rat (Goco, Kress & Brantigan, 1963), but different from the dog, which has a small number of submucosal glands (Goco, Kress & Brantigan, 1963), and the pig, which has a large number of submucosal glands and lymphoid cells in its tracheobronchial tree (Goco, Kress & Brantigan, 1963; Hartmann, Hutchison & Jewell, 1984; Lai-Fook & Hyatt, 1979).

The visceral pleura was composed of thick, dense fibrous connective tissue, with no obvious blood vessels (unlike the visceral pleura of the sheep). The parietal pleura was replaced by a layer of dense connective tissue overlying the intercostal muscles, with a layer of loose connective tissue outside (West, 2002) and intralung septa composed of thick fibroelastic tissue (Brown et al., 1997).

The epithelial lining of the bronchioles was composed of ciliated pseudostratified columnar epithelium with goblet cells, different from the horse which has simple columnar epithelium without mucin-containing goblet cells in its mucosa. Interestingly, thickening of fibroelastic tissues was observed around the bronchioles, similar to the horse (Goco, Kress & Brantigan, 1963).

Terminal bronchioles, respiratory bronchioles and alveolar ducts had thick walls of smooth muscle surrounded by collagen and elastin fibers, different from domestic animals (Brown et al., 1997), especially the dog and cat, whose alveolar walls have no fragmentation of elastic fibers or an increase in collagenous fibers (Leith, 1976; Loosli, Adams & Thornton, 1949). In addition, the microanatomy of the alveolar wall and alveoli were unlike the dog, which has lymphoid cells along the alveolar tissues (Goco, Kress & Brantigan, 1963).

### Gastrointestinal system

Microscopic examination of the alimentary or gastrointestinal tract of the juvenile Asian elephant showed some differences from other hindgut-fermentation animals such as the horse and rabbit, whose esophagus mucosa is generally a keratinized stratified squamous type, the degree of keratinized cells depending on the type of food ingested (Trautmann & Fiebiger, 1952). However, the elephant’s esophagus mucosa was non-keratinized stratified squamous epithelium (Van Aswegen et al., 1994). To protect the mucosa, the elephant excretes mucus from the plentiful submucosal mixed glands to lubricate the luminal surface of the esophagus. Skeletal muscle formed the tunica muscularis, as confirmed by identification of cross- and longitudinal striations; this differed from a study by Van Aswegen (Van Aswegen et al., 1994), who showed that the tunica muscularis contained visceral muscle. All of the anatomy and microanatomy of the elephant stomach was similar to the porcine stomach: a simple stomach with conical diverticulum ventriculi in the anterior border (Indu et al., 2014). The cardiac, fundic and pyloric regions consisted of branched, coiled and straight tubular glands, with chief cells and parietal cells. More chief cells and parietal cells were observed in the proximal parts of the stomach (cardia and fundus) than the distal parts (pylorus), as reported in laboratory mammals, such as mice, rat, hamsters, guinea pigs, gerbils and rabbits (Ghoshal & Bal, 1989). The elephant stomach has more gastric glands and parietal cells than the porcine stomach, in order to digest large amounts of coarse-fiber vegetation (Banks, 1993). Also, the thickness of the muscularis mucosae and tunica muscularis might provide additional strength to the wall during mechanical digestion of the large amount of food ingested. There were no lymphatic nodules in the tunica submucosa of elephant stomach sections, which was different from a previous report (Indu et al., 2014). The microanatomy of the small and large intestines of the elephant was similar to that of monogastric domestic animals (Banks, 1993). Brunner’s glands were prominent within the tunica submucosa of the duodenum, to produce digestive enzymes only in the proximal intestine. The location of Brunner’s glands is in the first two-thirds of the duodenum in dogs, cats and humans ((Banks, 1993) Lymphoid follicles were diffused in the lamina propria and tunica submucosa, especially in the colon, and were plentiful in the cecum. Goblet cells were plentiful, particularly in the intestinal epithelium and crypts in the large intestine related to hind-gut fermentation. The tunica muscularis was thick in most sections of the cecum and colon, which retain and ferment digesta. Peristalsis was observed in these portions of the intestine. Taeniae and haustra structures, which are characteristic gut segments in hind-gut fermentation animals, serve not only as a means to retain digesta, but also allow a significant distension of the corresponding gut section (Langer & Takács, 2004). It is common knowledge among horse owners that on roughage-dominated diets, horses show the typical “hay belly” after feeding; a similar phenomenon occurs in elephants (Clauss et al., 2007). The dorsal surface and lateral borders of the tongue were covered by a mucous membrane containing nerve endings for general sensory reception and taste perception. The dorsal surface of the tongue was covered with tiny projections called papillae, which were lacking on the ventral surface. The African elephant tongue also had 3–5 vallate papillae, foliate papillae in the posterolateral region, and filiform papillae distributed along the lateral border to the apex of tongue. Vallate and foliate papillae contained serous glands but had no taste buds (Kubota, 1967). No lingual papillae were observed on the dorsal surface of the tongue, and also no taste buds on the dorsal surface epithelium. This differed from the findings of another study, perhaps because this tongue was collected from a juvenile elephant. The architecture of the liver displayed normal organization, and did not differ to any great extent from the general mammalian structure. The pancreas tissues were autolytic and improperly fixed, so that exocrine and endocrine parts could not be identified.

### Urinary system

The renal capsules were composed of collagen and elastic fibers, which were thicker than in swine, equines and carnivores but similar to small ruminants (Maluf, 1995). The kidney of the juvenile Asian elephant had interlobar septa, fibromuscular fibers which separate the lobes of the kidney, no capillaries and large blood vessels. In contrast to the rhinoceros, the interlobar septa had interlobar arteries coursing through for blood supply (Maluf, 1989, 1994a, 1995).

Intrarenal vessels demonstrated no anastomoses between lobar, interlobular or intralobular arteries, similar to the dog (Arnautovic, 1959), sheep (Buys-Gonçalves et al., 2016) and camel (Jain & Gupta, 2000). In both Asian and African adult elephants, the glomerular diameter as a function of adult body mass is larger than in other mammalian species, such as the killer whale (*Orcinus orca*), human (*Homo sapiens sapiens*), horse (*Equus ferus caballus)*, hippopotamus (*Hippopotamus amphibius*), giraffe (Giraffidae spp.) and okapi (*Okapia johnstoni*) (Endo et al., 2002; Maluf & Gassman, 1998).

At the end of the renal papilla, the cribriform area or area cribrosa was flat or concave. In addition, the cribriform area can be inverted as a tubus maximus, which is an enlarged collecting duct (Maluf, 1995), similar to the pygmy hippopotamus (*Choeropsis liberiensis*) (Maluf, 1994b).

### Reproductive system

In this study, we have been unable to find a complete detailed description of the histology of reproductive system because they are juvenile elephants. The number of follicles in the ovary reserve of mammals is established during the development of the fetus and neonate. In Asian elephants, ovulation in each reproductive cycle is similar to that reported for African elephants (Stansfield, Nöthling & Allen, 2012). A number of follicles in an Asian elephant calf are recruited each day and become atretic follicles, while very few develop into the dominant follicle. The follicle reserve of the Asian elephant in this study was found to consist of early primary follicles (a single layer of cuboidal granulosa cells), true primary follicles (all cuboidal granulosa cells), and true primordial follicles (a single layer of squamous granulosa cells) grouped together as small follicles within the ovary. This is in agreement with previous studies on the African elephant, where the type of small follicles mostly found were early primary follicles and true primary follicles rather than primordial follicles (Stansfield, Nöthling & Allen, 2012; Stansfield, Nothling & Ansari, 2011a). Furthermore, the oocytes are larger in true primary follicles than in early primary follicles, suggesting activation of the follicle (Picton, 2001) and also that true primary follicles do not belong to the ovarian reserve of the small follicle pool in the African elephant (Stansfield, Nöthling & Allen, 2012) and possibly the Asian elephant as well. It is necessary to determine the number of small follicles, including primordial, early primary and true primary follicles, in the ovaries of elephants, as the follicle reserve supplies oocytes throughout the reproductive cycle (Stansfield, Nothling & Ansari, 2011a). The distribution of small follicles in the mammalian ovarian cortex is characterized as heterogeneous and species-specific (Charleston et al., 2007). The ovary of the African elephant has a lower density of small follicles per unit volume of ovarian cortex than other mammalian species (Stansfield, Picton & Nöthling, 2011b), including humans (Faddy et al., 1992), suggesting that it is important to certify that a sufficiently large sample of ovarian specimens is used for the assessment of the number of small follicles in the Asian elephant ovary. The young Asian elephant ovary contained interstitial tissue within the medulla, similar to that reported in African elephants (Stansfield, Nöthling & Allen, 2012), bovines (Van Wezel & Rodgers, 1996) and other mammals (Stansfield, Nöthling & Allen, 2012). There is a persistence of 3β-hydroxysteroid dehydrogenase (3βHSD)-positive nests of interstitial cells in the ovaries of female African elephant calves after birth (Stansfield, Nöthling & Allen, 2012). Further study is suggested to investigate the steroid secretion of these interstitial cells, as well as to determine 3βHSD staining of the granulosa cells of small follicles from ovarian specimens, and also the variation in the number of small follicles in the ovaries of Asian elephants. It is important to investigate follicle dynamics in the Asian elephant throughout its reproductive cycle.

The bicornuate uterus in the Asian elephant shows some similarity to that of the horse and the African elephant; however, the body of the elephant uterus is shorter than its counterpart in the horse (Allen et al., 2003), in contrast to the dromedary camel (Skidmore, Wooding & Allen, 1996). A transverse section across the uterus of an Asian elephant presented folding of the endometrium to form a star-shaped uterine lumen, as described previously in the African elephant (Allen et al., 2003). The endometrium showed lumenal epithelial layers within the lateral clefts of the star; the microscopic anatomy resembled the uterus of a rabbit more than other animal species (Allen et al., 2003). The epithelial lining of the endometrium in mammals varies from simple cuboidal or columnar (as in the horse, dog and cat) to stratified or pseudostratified (Aughey & Frye, 2001a). The epithelial tissue of the endometrium of the Asian elephant is lined by pseudostratified epithelium, which is similar to that of other related species, sows and ruminants (Aughey & Frye, 2001a). Future studies that include larger sample sizes of animal tissues and variations in the uterus may provide additional insights into the reproductive mechanisms of Asian elephants.

### Lymphatic system

Splenic capsules of thin smooth muscle and collagenous tissue were observed in the juvenile Asian elephant, whereas a thick fibromuscular capsule has been reported in the dog (Das et al., 2005). The splenic trabeculae of the elephant, containing smooth muscle cells, is similar to that of the sheep (Khalel, 2010). The trabeculae of the elephant and other mammals extend from the capsule into the parenchyma, whereas avascular trabeculae of the camel was documented (Zidan et al., 2000). The splenic parenchyma was comprised of a reticular meshwork arrangement of fibroblasts and extracellular matrix proteins, including fibronectin, laminin, vitronectin, tenascin, and type III and IV collagen (Liakka et al., 1995). The red pulp was the major compartment of the spleen (75%), which filters and degrades old or abnormal erythrocytes. The white pulp, mostly located at the central area of the parenchyma, enables punctual activation of leukocytes by foreign antigens. The white pulp of the Asian elephant was composed of three parts, including periarteriolar lymphoid sheaths, lymphoid follicles and the marginal zone. The marginal zone of the Asian elephant shows some similarity to that of cattle, dogs, mice and humans (Das et al., 2005; Vasilescu, 2011), whereas the absence of a germinal center in some lymphoid follicles has been reported in the ovine spleen (Khalel, 2010).

### Limitations of the study

Our study has some limitations. First, only two elephant calves were used as subjects. To confirm these microanatomical structures, additional animals are needed for study. Second, an adult elephant was not included in this study for comparison; however, we compared our findings with published data (when available) or with other species. Third, immunohistochemistry staining for specific cells in each tissue was not performed. And last, the length of time spent in tissue collection (12 h) resulted in some tissue damage or lysis. This took time because of the time required to transport the elephant carcass from the elephant camp to our facility.

## Conclusion

Here, we describe the normal histological in different tissues of an Asian elephant calf. Almost all structures were similar to those of other reported species or adult elephants. The sciatic nerve in elephants contains numerous fascicles and also have very thick epineurium. Skeletal system which represents plexiform bone (in long and flat bone) is only present in neonatal and young juvenile elephants. Moreover, we found that the long bone of adult elephants have double-zoned osteons. For the skin topic, the absence of both arrector pili muscles and sebaceous glands in elephants has already been reported. The thin trachealis muscle was observed in the trachea; and serous and mucinous glands were not found in the submucosa of trachea. The pleura and intralung septa were composed by thick, dense fibrous connective tissue. Terminal bronchioles, respiratory bronchioles and alveolar ducts have thick walls of smooth muscle surrounded by collagen and elastin fibers. We found that interlobar septa in kidney separated the lobes of the kidney. At the end of the renal papilla, the cribriform area could be inverted as a tubus maximus, which is an enlarged collecting duct. Elephant’s esophagus mucosa structure is a non-keratinized stratified squamous epithelium which is similar to other hindgut-fermentation animals. Regarding ovary in elephants, the ovarian cortex has lower density of small follicles per unit volume than other mammalian species. Splenic capsules of thin smooth muscle and collagenous tissue were observed in elephants. The histological structure of other structures such as the brain, cartilage, heart, blood vessel, muscle, stomach, intestine, liver, pancreas or uterus did not markedly differ from in the general mammalian structure.

Histological information from various organs can serve as an important foundation of basal data for future microanatomical studies, and help in the diagnosis and pathogenesis in sick elephants or those with an unknown cause of death. More samples from both sexes and various ages would enhance the microanatomical information for this species. Specific and functional stains, i.e., immunohistochemistry, could shed more light on the function of cells, tissues and organs.

## Supplemental Information

10.7717/peerj.4947/supp-1 Supplemental Information 1 A 2-year-old Asian elephant weighing 400 kg (A) and a 2-year and 9-month-old Asian elephant weighing 600 kg (B). Photograph by Chatchote Thitaram.

## Additional Information and Declarations

### Data Availability

The following information was supplied regarding data availability: The raw data is available in the Results section and the figures.
