# Supplementary figures and images for: Histology of 24 organs from Asian elephant calves (Elephas maximus)
Source: PeerJ. 2018 Jun 14;6:e4947. doi: 10.7717/peerj.4947 (PMC6004303; doi:10.7717/peerj.4947)

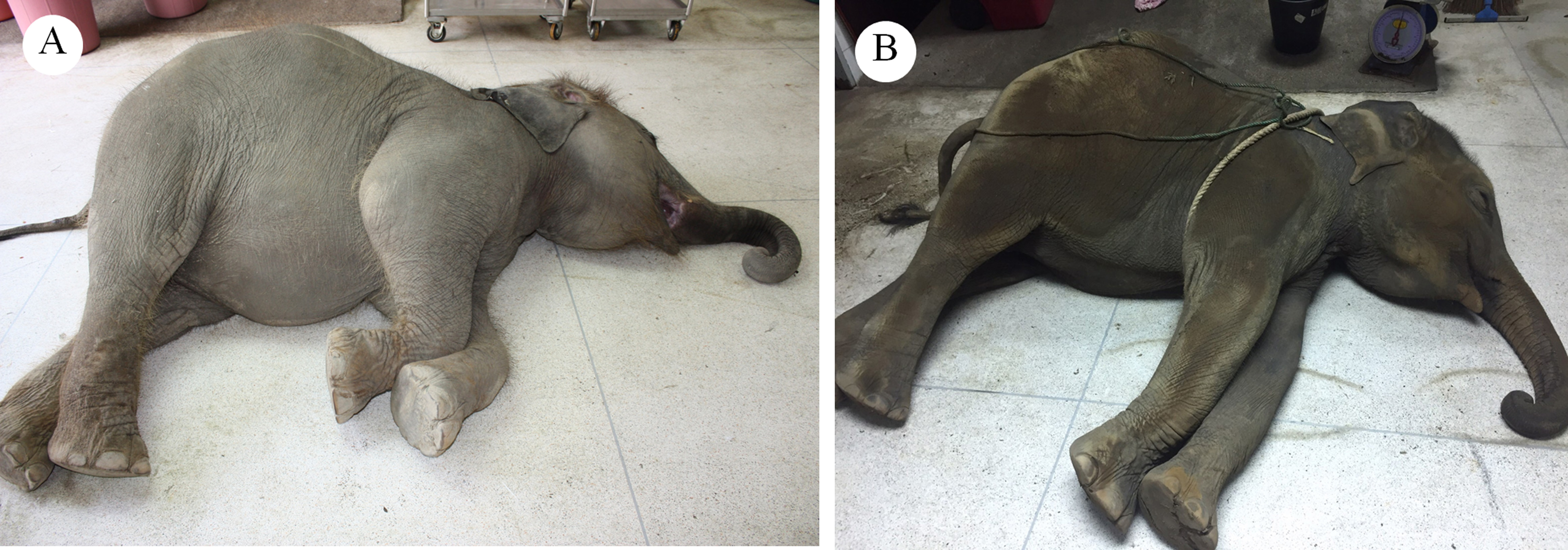

Supplement: Supplemental Information 1 [file peerj-06-4947-s001.jpg]
